# *Growth Factor Independence 1b* (*Gfi1b*) Is Important for the Maturation of Erythroid Cells and the Regulation of Embryonic Globin Expression

**DOI:** 10.1371/journal.pone.0096636

**Published:** 2014-05-06

**Authors:** Lothar Vassen, Hugues Beauchemin, Wafaa Lemsaddek, Joseph Krongold, Marie Trudel, Tarik Möröy

**Affiliations:** 1 Institut de Recherches Cliniques de Montréal, IRCM, Montréal, Québec, Canada; 2 Département de Microbiologie, Infectiologie et Immunologie, Université de Montréal, Montréal, Québec, Canada; 3 Division of Experimental Medicine, McGill University, Montréal, Québec, Canada; Southern Illinois University School of Medicine, United States of America

## Abstract

Growth factor independence 1b (GFI1B) is a DNA binding repressor of transcription with vital functions in hematopoiesis. *Gfi1b*-null embryos die at midgestation very likely due to defects in erythro- and megakaryopoiesis. To analyze the full functionality of *Gfi1b*, we used conditionally deficient mice that harbor floxed *Gfi1b* alleles and inducible (*Mx*-Cre, Cre-ERT) or erythroid specific (*EpoR*-Cre) Cre expressing transgenes. In contrast to the germline knockout, *EpoR*-Cre mediated erythroid specific ablation of *Gfi1b* allows full gestation, but causes perinatal lethality with very few mice surviving to adulthood. Both the embryonic deletion of *Gfi1b* by *EpoR*-Cre and the deletion in adult mice by *Mx*-Cre or Cre-ERT leads to reduced numbers of erythroid precursors, perturbed and delayed erythroid maturation, anemia and extramedullary erythropoiesis. Global expression analyses showed that the *Hba-x*, *Hbb-bh1* and *Hbb-y* embryonic globin genes were upregulated in *Gfi1b* deficient TER119^+^ fetal liver cells over the gestation period from day 12.5–17.5 p.c. and an increased level of *Hbb-bh1* and *Hbb-y* embryonic globin gene expression was even maintained in adult *Gfi1b* deficient mice. While the expression of *Bcl11a*, a regulator of embryonic globin expression was not affected by *Gfi1b* deficiency, the expression of *Gata1* was reduced and the expression of *Sox6*, also involved in globin switch, was almost entirely lost when *Gfi1b* was absent. These findings establish *Gfi1b* as a regulator of embryonic globin expression and embryonic and adult erythroid maturation.

## Introduction

The continuous process of hematopoiesis initiating from pluripotent hematopoietic stem cells and giving rise to all hematopoietic lineages compensates for the restricted life span of mature blood cells. Each terminally differentiated blood cell is the result of chronological steps of proliferation and differentiation, which are stringently controlled by underlying lineage specific and ubiquitously expressed transcription factors. The DNA binding repressors of transcription growth factor independence 1b (GFI1B) and its paralogue GFI1 are expressed in a complementary and partially overlapping manner in hematopoietic stem cells and several hematopoietic lineages as well as cells of the sensory and nervous systems [1]–[3]. Although knockout mutants for both proteins in mice resulted in different hematopoietic phenotypes [4]–[11], GFI1B can functionally replace GFI1 throughout the hematopoietic system, but not in sensory cells such as the inner ear hair cells [2].

Both Gfi1 and *Gfi1b* are considered to be proto-oncogenes and have been linked to several hematologic malignancies [5], [12]–[23], underscoring the importance of their adequate regulation during blood cell differentiation. *Gfi1b* is expressed in hematopoietic stem cells (HSC), myeloid/erythroid precursors (MEP), megakaryocytes and to varying levels during erythrocyte maturation [1]. Accordingly, these are the cell-types with the most obvious phenotype in *Gfi1b* knockout mice and GFI1B has been described as an essential factor in embryonic erythroid and megakaryocytic development [6], [24]–[26]. The expression of *Gfi1b* is subject to autoregulation and crossrepression by Gfi1 [27], [28]. Expression of *Gfi1b* in the erythroid lineage is controlled by GATA1, to which GFI1B can bind, by NF-Y in K562 cells [29] and by HMGB2 in human erythroid differentiation [30]. The GFI1B/GATA1 complex is also involved in the auto-regulation of *Gfi1b* [31]–[33]. The expression of *Gfi1b* is downregulated by *Oct1* and upon erythropoietin signaling in a *Stat5* dependent manner [34], [35].

Repression of transcription by *Gfi1* or *Gfi1b* fully depends on its N-terminal Snail/Gfi (SNAG) domain, which enables the recruitment of the GFI1/GFI1B cofactors Lysine (K)-specific demethylase 1A (LSD1/KDM1A) and CoREST/Rcor1. Consequently, a knockdown of LSD1 has been shown to cause a phenotype reminiscent of *Gfi1b* or *Gfi1* knockout phenotypes affecting HSCs, granulopoiesis, erythropoiesis and platelet production [36]. The function of the GFI1B/LSD1/CoREST complex in erythroid proliferation and differentiation was intensively studied [37], [38]. Interestingly, the GFI1B/LSD1/CoREST complex binds to the *Meis1* promoter in erythroid cells, but not in megakaryocytes, despite the fact that it is highly expressed in both cell types, suggesting a functional difference of *Gfi1b* between the two lineages.

Germline deletion of *Gfi1b* in mice causes lethality at around day 14.5 of embryonic development, probably due to a combined phenotype of inappropriate erythropoiesis and severe bleeding caused by a failure to produce platelet-generating megakaryocytes [1], [6]. However, other not yet discovered mechanisms may also play a role. This early lethality of *Gfi1b* deficient mice restricted all analyses to either prenatal hematopoiesis or to cell culture systems. The recent generation of conditional *Gfi1b* knockout mice [9] allowed us to perform a more specific analysis of pre- and postnatal function of *Gfi1b* in erythropoiesis. We inactivated the *Gfi1b* gene by crossing conditional *Gfi1b* knockout mice with *EpoR*-Cre knock-in mice to delete specifically in the erythroid lineage or by inducibly ablating it in adult mice using *Mx*-Cre and *Rosa*-Cre-ERT mouse lines. Our results show that *Gfi1b* is required for the differentiation from pro-erythroblasts to mature erythrocytes and for the silencing of globin genes during embryonic development and at adult stages.

## Methods

### Ethics Statement

The protocols for the in vivo experiments described here were reviewed and approved by the IRCM Animal Care Committee (ACC); protocol numbers are: #2009-12/#2013-04. All animal experiments were conducted according to institutional rules put in place by the IRCM ACC, which follow the regulations and requirements of the Canadian Council on Animal Care (www.ccac.ca).

### Mice

The generation of *Gfi1b^−^* ^GFP^ knock-in mice and *Gfi1b* ^fl/fl^ conditional knockout mice has been described previously [1], [9]. All mice were housed under specific pathogen-free conditions and institutional animal ethics committees reviewed animal experimentation protocols and certified animal technicians regularly observed the mice in sign of distress. Adult mice were sacrificed by carbon dioxide inhalation whereas newborn pups were euthanized by decapitation following anesthesia by carbon dioxide inhalation as per standard operating procedure approved by the IRCM ACC and the CCAC. All efforts were made to minimize the number of animals used and to reduce their suffering. All mice were backcrossed with C57BL/6 mice for at least 8 generations. No phenotype or differences in number of cells was observed for *Gfi1b* ^fl/fl^ or *Gfi1b* ^KO/fl^ mice. *Rosa*-Cre-ERT mice were obtained from The Jackson Laboratory (Bar Harbor, Maine, USA), strain B6;129-Gt(*Rosa*)26Sortm1(cre/ERT)Nat/J Stock Nr. 004847. The generation of *EpoR*-Cre knock-in mice used for erythroid specific ablation of *Gfi1b* expression has been described previously [39].

### Flow cytometry, cell sorting, microarray analysis and Q-PCR

Hematopoietic cell populations were analyzed by flow cytometry using an LSR (BD Biosciences) and sorted using a MoFlo (Cytomation). Cells were passed through a 23-gauge needle, filtered through a cell strainer and resuspended in PBS (1% FCS, 10 mM EDTA). 1-5 X 10^6^ cells were stained with antibodies at a 1∶200 concentration for 20 min, washed with PBS and measured or sorted immediately. Antibodies used were ordered from BD-Biosciences (Missisauga, ON, Canada) or Bio-Legend (San Diego, CA, USA).

TRIzol (Invitrogen) was applied to isolate RNA/DNA/protein from sorted cells according to the manufacturers protocol. Quantitative RT-PCR was performed in a 20 µl reaction volume containing 900 nM of each primer, 250 nM TaqMan probe, and 1 µl TaqMan Universal PCR Master Mix (ABI, Germany) according to the manufacturer's instructions. The relative expression of genes of interest was calculated relative to the GAPDH mRNA levels. Primers used for quantitative analysis of mRNA were: m_alpha-F: gggtcacggcaagaaggt; m_alpha-R: tgctcacagaggcaaggaat; β-min-maj-ex2-F: tttaacgatggcctgaatcactt; β-min-maj-ex3-R: cagcacaatcacgatcatattgc; ey-ex1-F: tggcctgtggagtaaggtcaa; ey-ex2-R: gaagcagaggacaagttccca; β-h1-ex2-F: tggacaacctcaaggagacc; β-h1-ex3-R: acctctggggtgaattcctt; Hba-x-F: cgggcccacggcttcaagat; Hba-x-R: caggggtgaagtcggcggga; mBcl11a-F: gcacttaagcaaacgggaat; mBcl11a-R: caggtgagaaggtcgtggtc; mSox6-F: aatttggacccctctgaaca; mSox6LS: agctgagcggcatagagc; *Gfi1b*-ex3-4-F: ccagaccttggactggaaca; *Gfi1b*-ex3-4-R: ggagaagctgggcttgtaga; mGata1-F: gaatcctctgcatcaacaagc; mGata1-R: gggcaagggttctgaggt;

Primers used for genotyping were: *Gfi1b* allele: LP5-3s: ggtttctaccagtctggccctgaactc; LP5-3r: ctcacctctctgtggcagtttcctatc; LP5-4r: tacattcatgcttagaaacttgagtc; product length of the different alleles is: wt, 256 bp; floxed, 295 bp and deleted, 540 bp. Internal control: mRag1.1: gctgatgggaagtcaagcgac; mRag1.3: gggaactgctgaactttctgtg. *EpoR*-Cre: 06-44: gtgtggctgccccttctgcca; 06-45: ggcagcctgggcaccttcac; 06-46: caggaattcaagctcaacctcaFor whole genome gene expression analysis of TER119^+^ wild type or *Mx*-Cre induced *Gfi1b*-KO erythroid cells from adult mice, a total of 10 µg cRNA from sorted cells was hybridized on Affymetrix (Affymetrix Inc., Santa Clara, CA, USA) Mouse Genome 430A 2.0 arrays (GPL1261). For analysis of wild type or *EpoR*-Cre induced TER119^+^ *Gfi1b*-KO fetal liver cells at 14.5 dpc, cRNA was hybridized to Affymetrix mouse Gene 1.0 ST arrays (GPL6246). Microarray data have been deposited in the public database Gene Expression Omnibus (National Center for Biotechnology Information; GEO accession number: GSE54206). Data were analyzed with AltAnalyze [40] software using the default settings. Where given in the figures or text, the rawp is a one-way analysis of variance (ANOVA) p-value calculated for each pairwise comparison (two groups only). The log-fold is the log2 fold calculated by geometric subtraction of the experimental from the control groups for each pairwise comparison. Gene set enrichment analysis was performed using the GSEA software (www.broadinstitute.org/gsea) [41]. The nominal p value (n. p-val) given for most analyses estimates the statistical significance of the enrichment score for a single gene set. Given 1000 gene-set permutations we chose for each analysis, a p value of zero (0.0) indicates an actual p value of less than 0.0001. Hierarchical clustering analysis and scatter plots were generated using Spotfire Decision Site for functional genomics software (www.spotfire.tibco.com). Statistical analysis: The unpaired Student's t-test was chosen for analyzing data distribution.

## Results

### Erythroid specific ablation of *Gfi1b* by *EpoR*-Cre causes accumulation of immature erythroid cells and perinatal lethality

**Figure 1:**
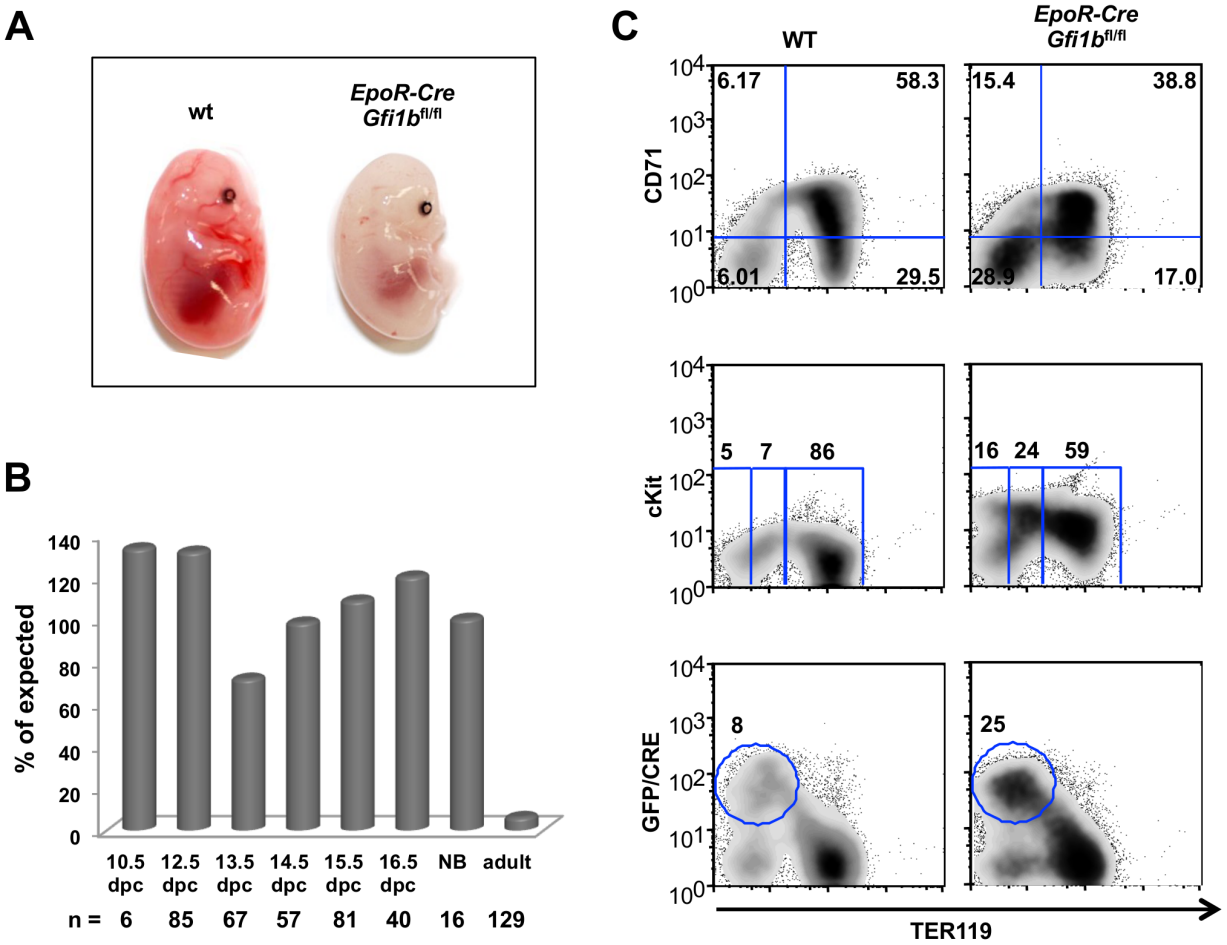
The erythroid specific knockout of *Gfi1b* causes perinatal lethality and a delayed maturation of fetal erythroid cells. A: Typical situs of either wild type (wt) or specific knockout of *Gfi1b* in the erythroid lineage (*EpoR*-Cre, *Gfi1b* ^fl/fl^). While germline deletion of *Gfi1b* results in anemic, pale embryos with severe hemorrhage, the erythroid lineage specific knockout of *Gfi1b* results in pale embryos but not in hemorraghe (right). B: Bar graph illustrating the percentage of expected *EpoR*-Cre transgenic *Gfi1b* ^fl/fl^ embryos found viable at different stages of development as indicated. The erythroid specific ablation of *Gfi1b* does not reduce the survival rates of mouse embryos until birth, but leads to perinatal lethality with few exceptions (two out of 129 at age 6-weeks). The total numbers of embryos (N) analyzed at each stage are indicated at the bottom. Pregnant females were humanly euthanized according to procedures approved by the Canadian Council on Animal Care (CCAC) at indicated gestational time points and embryos were taken for analysis. New born pups that survived until a few hours after birth were examined as soon as possible for signs of anemia (paleness), weakness and difficulty breathing (endpoints) and those showing such signs were humanly euthanized for analysis following procedures approved by the Canadian Council on Animal Care (CCAC). The two mice that survived to adulthood were monitored semiweekly but never showed any sign of distress and were perfectly healthy. These mice were humanly euthanized after 6 weeks for analysis. C: Flow cytometry of isolated fetal liver cells at 14.5 dpc from wt and erythroid specific *Gfi1b*-KO embryos using antibodies against TER119 in combination with the developmental markers CD71, cKIT and CD9 as well as GFP for the detection of the GFP-CRE fusion protein. GFP-Cre is expressed early in erythroid development before cells become TER119^+^ (TER119/GFP-CRE panel). *Gfi1b*-KO embryos show an accumulation of TER119-low, CD71-low, cKIT-high, CD9-high erythroid precursors indicating a delayed maturation of erythrocytes. FACS plots are representative for at least 6 or more independent samples analyzed.

We generated a cell type specific knockout of *Gfi1b* using *EpoR*-Cre mice, which express a GFP-Cre recombinase fusion protein in immature erythroid cells (Figure 1A, Figure S1), which also allows monitoring the expression of the Cre-transgene by measuring green fluorescence. *EpoR*-Cre *Gfi1b* ^fl/fl^ mice did not show internal bleeding at stage E14.5 (Figure 1A) or at birth, but appeared pale compared to controls (Figure 1A, Figure S1A). Most *EpoR*-Cre *Gfi1b* ^fl/fl^ mice died within minutes after birth, but a few survived to adulthood (Figure 1B, Figure S1A). *EpoR*-Cre *Gfi1b* ^fl/fl^ fetal livers (E14.5) appeared similar to wild type controls (Figure S1C), but showed an accumulation of CD71^−^ TER119^−^ erythroid precursor cells that are c-Kit^+^ and GFP^+^ (i.e. express the *EpoR*-Cre transgene) and a decrease of CD71^+^ TER119^+^ erythroblasts (Figure 1C), suggesting that deletion of *Gfi1b* delays the differentiation of embryonic erythroid cells.

### Acute disruption of *Gfi1b* in adult mice affects erythroid differentiation and causes anemia

**Figure 2:**
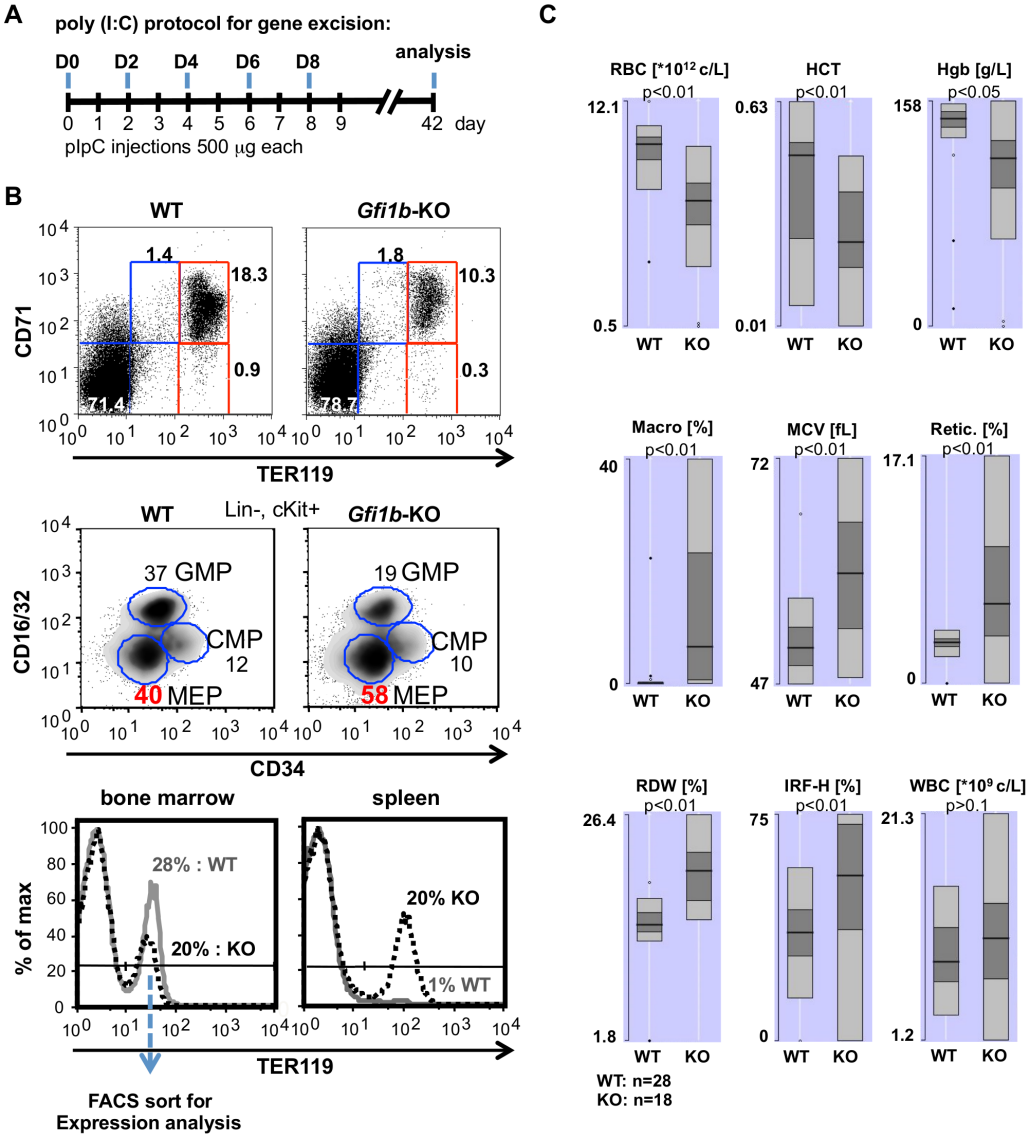
*Gfi1b* regulates maturation of erythroid cells in adult mice. A: *Gfi1b* ^fl/fl^ mice either carrying (*Gfi1b*-KO) the *Mx*-Cre transgene or not (wild type) were treated five times with pIpC (500 µg each) every other day and sacrificed for analysis 42 days after the first injection. B: Flow cytometric analysis of progenitors (middle panel) and maturing erythrocytes from the bone marrow (upper and lower panel) and spleen (lower panel) of wild type and *Gfi1b*-KO mice. TER119^+^ bone marrow cells from pIpC induced wild type and *Gfi1b* knockout animals were isolated by flow cytometry and RNA was prepared for microarray analysis of gene expression as indicated (lower left panel). FACS plots are representative for at least four individual samples from each genotype. C: Peripheral blood of wild type and conditional *Gfi1b*-KO mice was analyzed using an ADVIA hematology system and the comparison results are presented as box-whisker plots showing the central location and distribution of the indicated measures. Red blood cell count (RBC), hematocrit (HCT), hemoglobin (HGB), macrocytic RBCs (Macro), mean corpuscular volume (MCV), reticulocytes (Retic), red cell size and shape (RDW), immature reticulocytes fraction high (IRF-H) and white blood cell count (WBC).

To investigate the role of *Gfi1b* in adult erythroid development, we used *Gfi1b* ^fl/fl^ mice expressing the inducible *Mx*-Cre transgene [42] and injected them with pIpC (Figure 2A) to induce the recombination of the *Gfi1b* alleles throughout the hematopoietic system. Deletion of the floxed *Gfi1b* alleles in bone marrow, spleen and in FACS sorted TER119^+^ cells was substantial but still incomplete (Figure S2). Nonetheless, *Gfi1b* knockout mice showed a relative increase of MEP percentage over GMPs and CMPs in bone marrow compared to controls (Figure 2B, middle panel). Also, the proportion of CD71^+^, TER119^+^ pro-erythroblasts and of more mature CD71^−^, TER119^+^ erythroblasts was decreased (Figure 2B, upper panel), indicating a delay in erythroid maturation similar as seen in *EpoR*-Cre, *Gfi1b* ^fl/fl^ mice. Spleens of *Mx*-Cre, *Gfi1b* ^fl/fl^ mice were larger in size and showed a significant increase of TER119^+^ cells, suggesting ongoing extramedullary erythropoiesis (Figure 2B, lower panel and Figure S3). Peripheral blood analysis revealed a significant decrease in red blood cell count (RBC), hematocrit (HCT) and hemoglobin (Hgb) in *Gfi1b* deficient mice compared to wild type controls (Figure 2C). Consequently, numbers of reticulocytes (Retic), immature reticulocytes (IRF-H) and macrocytic RBCs (Macro) were increased in the absence of *Gfi1b* as well as the mean corpuscular volume (MCV) and red cell size and shape (RDW), while the white blood cell count (WBC) did not change significantly (Figure 2C), indicating that *Gfi1b* deficient mice suffer from anemia.

**Figure 3:**
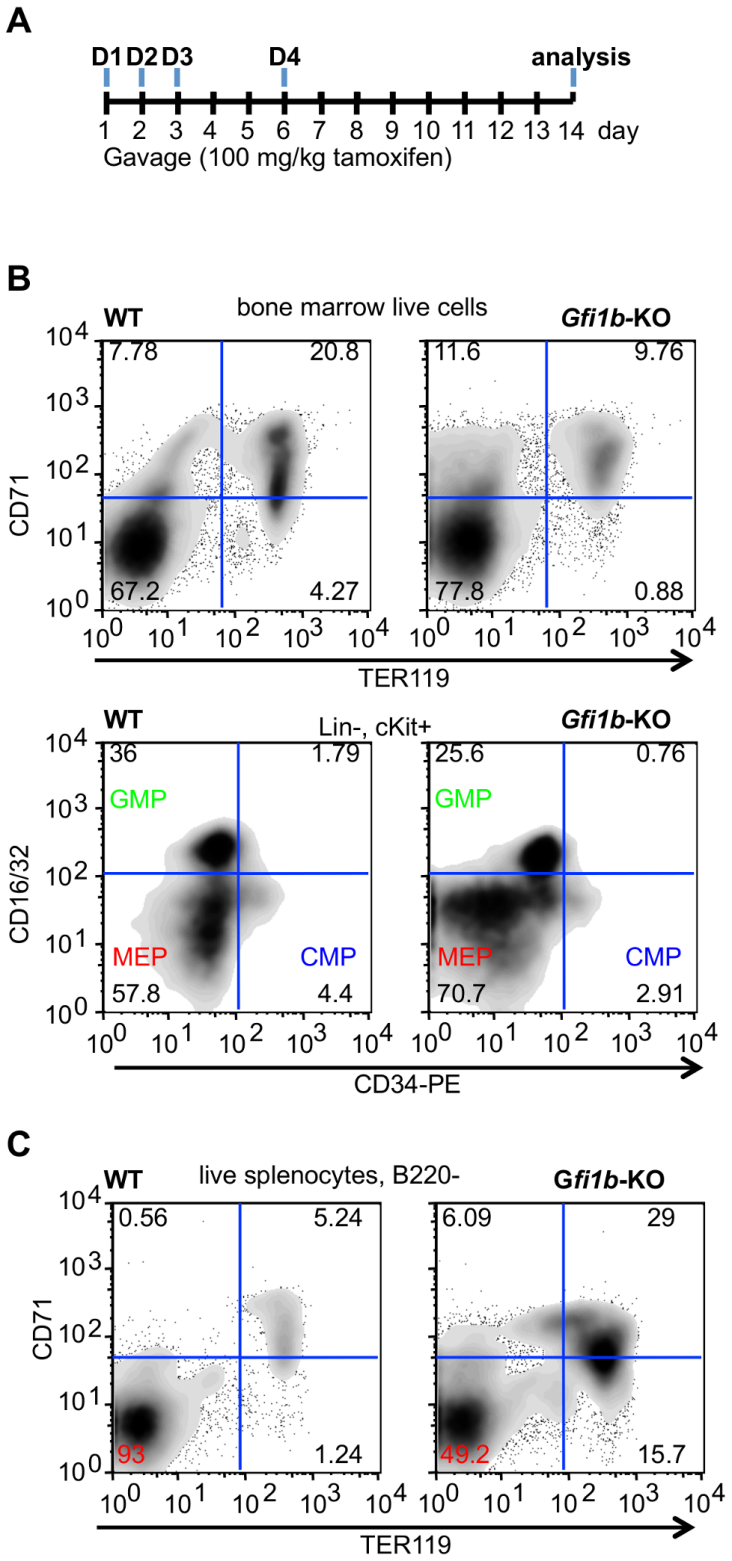
Ablation of *Gfi1b* in adult *Rosa*-Cre-ERT, *Gfi1b* ^GFP/fl^ mice by tamoxifen confirms defects in erythroid cell fate decision and maturation causing compensated anemia. A: Protocol used to efficiently generate adult *Gfi1b*-KO mice using tamoxifen. Eight week old *Gfi1b* ^fl/GFP^ mice either carrying a Cre-ERT knock-in into the *Rosa*26 locus (inducible *Gfi1b*-KO) or not (wt control) were treated by gavage with tamoxifen (100 mg/kg body weight) freshly dissolved in corn-oil at day 0, 1, 2 and 5 and sacrificed for analysis at day 14 of the experiment. B: Flow cytometric analysis of total bone marrow cells or FACS sorted Lin^−^, cKit^+^ cells from wild type and *Gfi1b*-KO mice for the presence of the indicated markers (TER119, CD71, CD34 and CD16/32. C: Flow cytometric analysis of stress erythropoiesis in the spleen using cells from wild type (left panel) or *Gfi1b*-KO (right panel) mice. FACS plots from (B) and (C) are representative for three or more individual samples from each genotype.

To further confirm these findings, we used mice carrying a tamoxifen inducible Cre recombinase integrated into the *Rosa26* locus for the ablation of *Gfi1b* expression in adult mice. In these mice the recombination of one or both conditional *Gfi1b* alleles was still incomplete when both *Gfi1b* alleles were floxed, but was efficient when only one floxed allele was present (Figure S4). We therefore used mice in which one floxed allele was replaced by a *Gfi1b*∶GFP knock-in allele, which disrupts the *Gfi1b* coding region and allows to measure *Gfi1b* mRNA expression by monitoring green fluorescence [1]–[3]. *Rosa*-Cre-ERT, *Gfi1b* ^GFP/fl^ mice were sacrificed between eight and 15 days after the last of four treatments with tamoxifen (Figure 3A). Flow cytometric analysis of bone marrow cells from these mice showed strongly reduced percentages of late erythroblasts (TER119^+^, CD71^+^ and TER119^+^, CD71^−^ cells), a slight increase of pro-erythroblasts (TER119^−^, CD71^+^ cells) and a marked increase of the percentage of MEPs at the expense of GMPs compared to controls (Figure 3B). The spleen in *Rosa*-Cre-ERT, *Gfi1b* ^GFP/fl^ mice was enlarged about twofold (not shown) and showed a significant accumulation of TER119^+^ cells (Figure 3C), which is indicative for ongoing extramedullary erythropoiesis as was also seen in *Mx*-Cre, *Gfi1b* ^fl/fl^ mice. This suggests that *Gfi1b* deficiency initiates a mechanism to compensate for marrow insufficiency. In addition, *Rosa*-Cre-ERT, *Gfi1b* ^fl/fl^ mice showed similar alterations in their blood parameters as *Gfi1b* ^fl/fl^- *Mx*Cre animals, namely reduced red blood cell count (RBC), hematocrit (HCT) and hemoglobin (Hgb) and increased numbers of reticulocytes, indicating again an anemic state in the absence of *Gfi1b* (not shown).

To gain more insight into the effects of tamoxifen on more differentiated erythroblast populations, we analyzed bone marrow of *Rosa*-Cre-ERT, *Gfi1b* ^GFP/fl^ and *Rosa*-Cre-ERT, *Gfi1b* ^GFP/+^animals from day 2 to day 8 after two tamoxifen injections using gates that divide TER119^+^ cells into proerythroblasts, basophilic erythroblasts, polychromatophilic erythroblasts and orthochromatophilic erythroblasts. Between day 2 and day 5 after tamoxifen injection, both basophilic and polychromatophilic erythroblasts almost entirely disappeared regardless whether a *Gfi1b* allele remained intact or not (Figure S5). Between day 6 and day 8 after tamoxifen injection, basophilic erythroblasts became detectable in the control mice but not or only to a lower extent in the Gf1b deleted animals (Figure S5). This suggests that Cre-ERT has a detrimental effect on TER119^+^ cells upon tamoxifen treatment and that these cells require *Gfi1b* for the differentiation of the TER119^−^ precursor population.

To test the long term effect of *Gfi1b* ablation by activating Cre-ERT, *Rosa*-Cre-ERT, *Gfi1b* ^GFP/+^ and *Rosa*-Cre-ERT, *Gfi1b* ^GFP/fl^ mice were analyzed two or nine months after tamoxifen-induced deletion of *Gfi1b*. TER119^+^ cells were gated again into four erythroblast populations. While a decrease of basophilic erythroblasts was still maintained, all other TER119^+^ cells were present at wt frequencies at both nine months (data not shown) and two months after tamoxifen induced *Gfi1b* ablation (Figure S6A, B). In animals with floxed *Gfi1b* alleles, only TER119^−^ cells showed efficient Cre mediated excision two months after tamoxifen induction, whereas the TER119^+^ erythroblast population only contained floxed or GFP alleles (Figure S6C). This indicated that these TER119^+^ erythroblasts very likely emerged from few non-deleted precursors in the TER119^−^ population, which supersede those with efficient excision of the *Gfi1b* allele and develop into TER119^+^ erythroid cells.

### *Gfi1b* deficient cells shows defects in the regulation of globin gene expression

**Figure 4:**
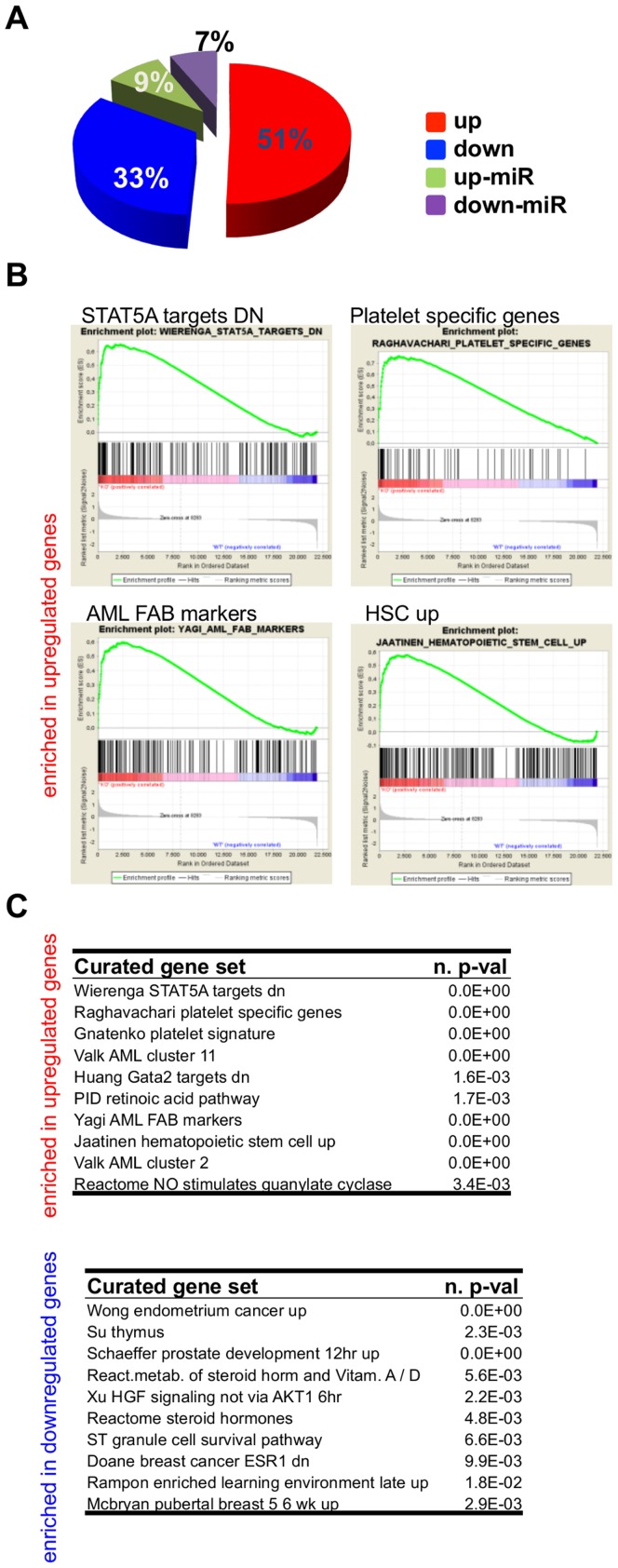
Loss of *Gfi1b* affects the expression of STAT5 target genes and megakaryocyte/platelet genes in bone marrow derived TER119^+^ cells. A: Pie chart of the statistical analysis of the numbers of protein coding genes regulated up or down more than two-fold in erythroid cells of *Mx*-Cre induced adult *Gfi1b* knockout mice. B: Gene set enrichment analysis (GSEA) comparing expression profiles of wild type and *Gfi1b* deficient TER119^+^ cells reveals a significant enrichment of genes up-regulated in *Gfi1b* deficient cells that are targets of Stat5 signaling, marker genes for megakaryocytes/platelets, marker genes for AML or show a high expression in hematopoietic stem cells (HSC). C: Tables are showing the 10 most significant results of GSEA as in (B) using curated genesets from the Molecular Signatures Database (MSigDB) either for genes that are upregulated (upper table) or downregulated (lower table) in *Gfi1b* deficient mouse bone marrow derived TER119^+^ cells.

**Figure 5:**
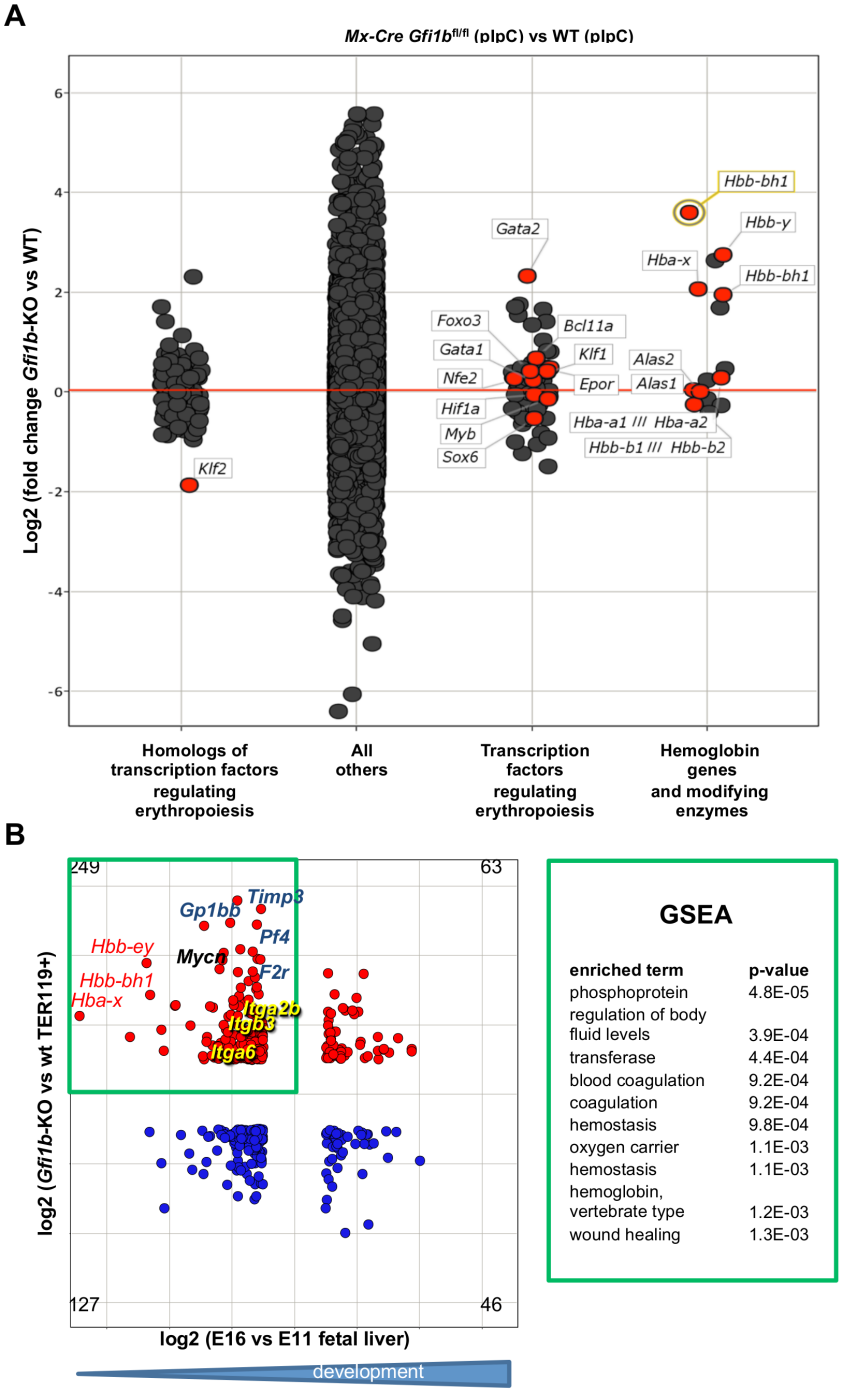
Deregulation of embryonic globin genes in *Gfi1b* deficient TER119^+^ cells. A: Scatter plot comparison of gene expression levels (log2 of normalized signal intensities) in TER119^+^ pIpC induced *Mx*-Cre, *Gfi1b* ^fl/fl^ bone marrow cells compared to pIpC induced cells from control mice. Dots represent probesets and are jittered for better visualization. Probesets were classified as indicated and probesets for hemoglobin genes and important regulators of hemoglobin gene expression and globin switch were labeled (red dots). Two RNA samples for each genotype were pooled and analyzed on single arrays. B: Scatter plot comparing the changes in gene expression induced by inactivation of *Gfi1b* in erythroid cells of adult mice (y-axis) as in (A) with genes regulated in fetal liver cells during development of the mouse embryo from 11 dpc (E11) compared to 16 dpc (E16). Raw data for fetal liver development was taken from GEO data series GSE13149 and reanalyzed. Embryonic globin genes (red), megakaryocyte/platelet specific genes (blue) and integrins known to be targets of *Gfi1b* in hematopoietic stem cells (yellow) are indicated. Genes that are downregulated in fetal liver cells during mouse development but upregulated in *Gfi1b*-KO cells are likely to be direct targets of the transcriptional repressor *Gfi1b* and were subjected to GSEA analysis (green frame and table to the right) and show a high enrichment in megakaryocytic/coagulation related genes and globin genes.

To gain more insight into the maturation defect caused by *Gfi1b* deficiency and to avoid any non-specific effects seen with tamoxifen, we performed two independent genome wide expression profiling experiments with FACS-sorted TER119^+^ bone marrow cells from *Gfi1b* ^fl/fl^, *Mx*-Cre mice (see Figure 2B, lower panel for sorting gate) and from TER119^+^ fetal liver cells from *EpoR*-Cre *Gfi1b* ^fl/fl^ mice. Most of the significantly regulated protein coding genes were upregulated in *Mx*-Cre/pIpC induced *Gfi1b* deficient cells compared to controls, which is in agreement with the role for *Gfi1b* as a transcriptional repressor (Figure 4A). Gene set enrichment analysis (GSEA) revealed targets of Gata2 and genes negatively regulated by Stat5 to be most affected by *Gfi1b* deficiency (Figure 4B, C). Platelet, HSC and AML specific genes were also significantly enriched among *Gfi1b* effector genes, which were up regulated in *Gfi1b* deficient cells (Figure 4B). Analysis of factors known to be associated with erythropoiesis showed that the expression of Gata2, Klf2, Bcl11a, Sox6 and the embryonic globin genes Hba-x, Hbb-bh1 and Hbb-y were affected by the deletion of *Gfi1b* (Figure 5A). A comparison of genes up-regulated in *Gfi1b* deficient TER119^+^ erythroid cells with those down-regulated during embryonic development revealed that expression of the embryonic globin genes *Hba-x*, *Hbb-bh1* and *Hbb-y*was strongly affected by *Gfi1b* ablation (Figure 5B). In addition, integrins alpha6 and alpha2b/b3 (CD41/61), already described as *Gfi1b* effectors in HSCs [9] and several other megakaryocyte/platelet specific genes such as *Gp1bb* (CD42C), *Timp3* or *Pf4* showed increased expression in *Gfi1b* deficient cells (Figure 5B).

**Figure 6:**
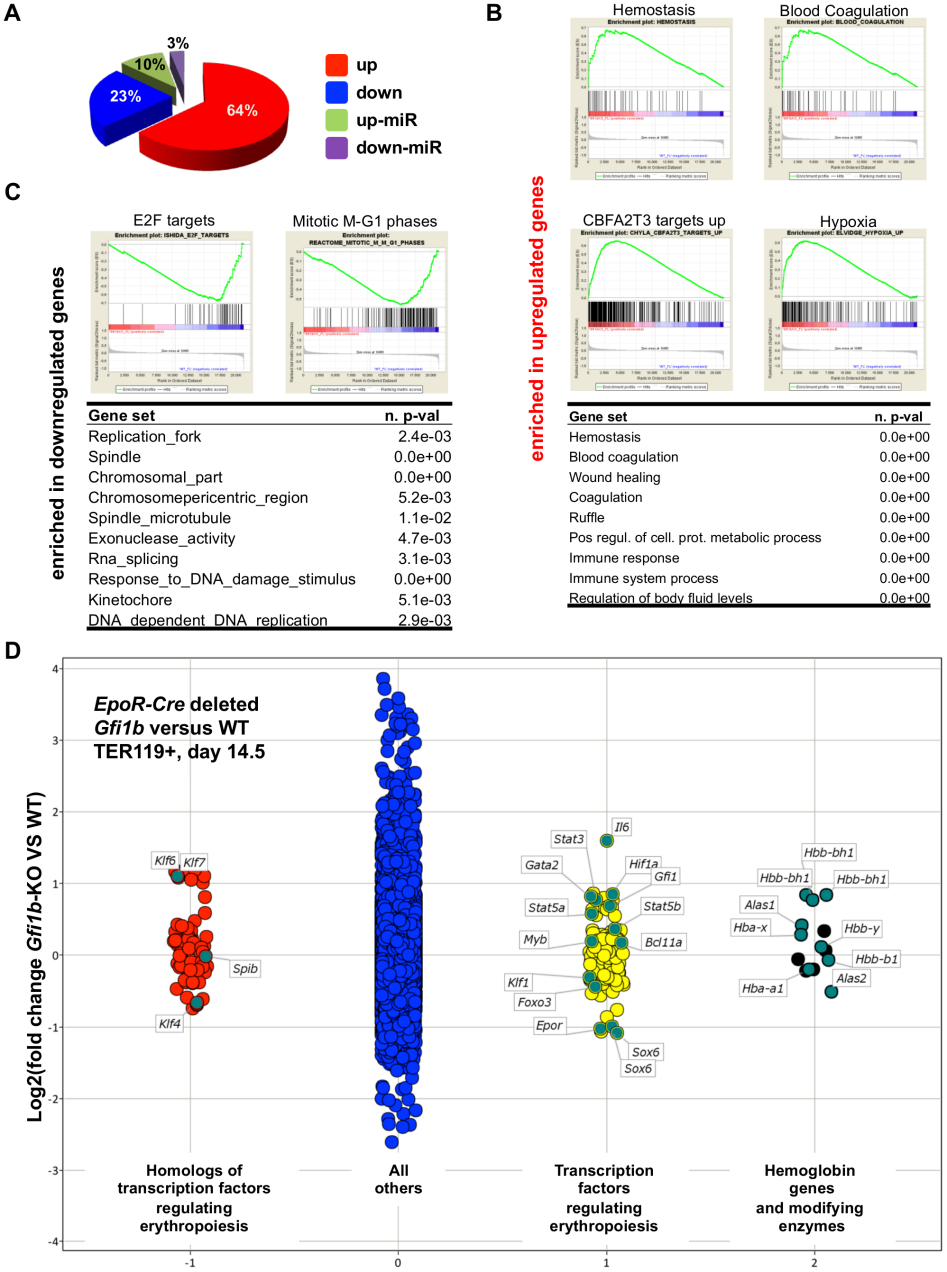
Transcriptome analysis of TER119^+^ fetal liver cell derived microarray data. A: Pie chart visualization of the proportion of genes up and down regulated more than two-fold in *EpoR*-Cre induced *Gfi1b* deficient fetal liver cells at 14.5dpc derived from microarray analysis. Array analysis for each genotype was done in duplicates. B: Gene set enrichment analysis shows enrichment in genes upregulated in *Gfi1b* deficient fetal liver cells related to hemostasis/coagulation, hypoxia and targets of the transcriptional corepressor Cbfa2t3 (upper enrichment plots and table). C: Cell division related gene sets and targets of the master regulator of replication E2F are enriched in downregulated genes in the *Gfi1b* knockout. D: Scatter plot comparison of gene expression levels (log2 of normalized signal intensities) in TER119^+^ cells from *EpoR*-Cre induced *Gfi1b* deficient fetal liver cells at 14.5dpc compared to the respective wild type cells. Dots represent probesets and are jittered for better visualization. Probesets were classified as indicated and probesets for hemoglobin genes and important regulators of hemoglobin gene expression and globin switch were labeled.

The second expression profiling experiment comparing mRNA prepared from TER119^+^ fetal liver cells from *EpoR*-Cre, *Gfi1b* ^fl/fl^ mice and wild type littermates isolated at 14.5 dpc also demonstrated that over 60% of protein coding genes whose expression changed more than two-fold were up-regulated in *Gfi1b* knockout cells, which was again in agreement with a repressor function of *Gfi1b* (Figure 6A, pie diagram). Exon analysis confirmed deletion of the targeted *Gfi1b* exons in the TER119^+^ fetal liver cells used for the analysis (Figure S7). GSEA analysis revealed that gene sets related to coagulation and immune response, hypoxia and CBFA2T3 (*Eto2*) targets were enriched among up-regulated genes, whereas gene sets related to cell cycle regulation, targets of E2F and DNA synthesis were enriched among down-regulated genes (Figure 6B, C). Similar to the analysis of TER119^+^ bone marrow cells from *Mx*-Cre, *Gfi1b* ^fl/fl^ mice, a deregulated expression of genes encoding GATA2, SOX6 and of the *Hba-x* and *Hbb-bh1* embryonic globin genes was observed (Figure 6D).

### *Gfi1b* is required for the developmental repression of embryonic globin gene expression

**Figure 7:**
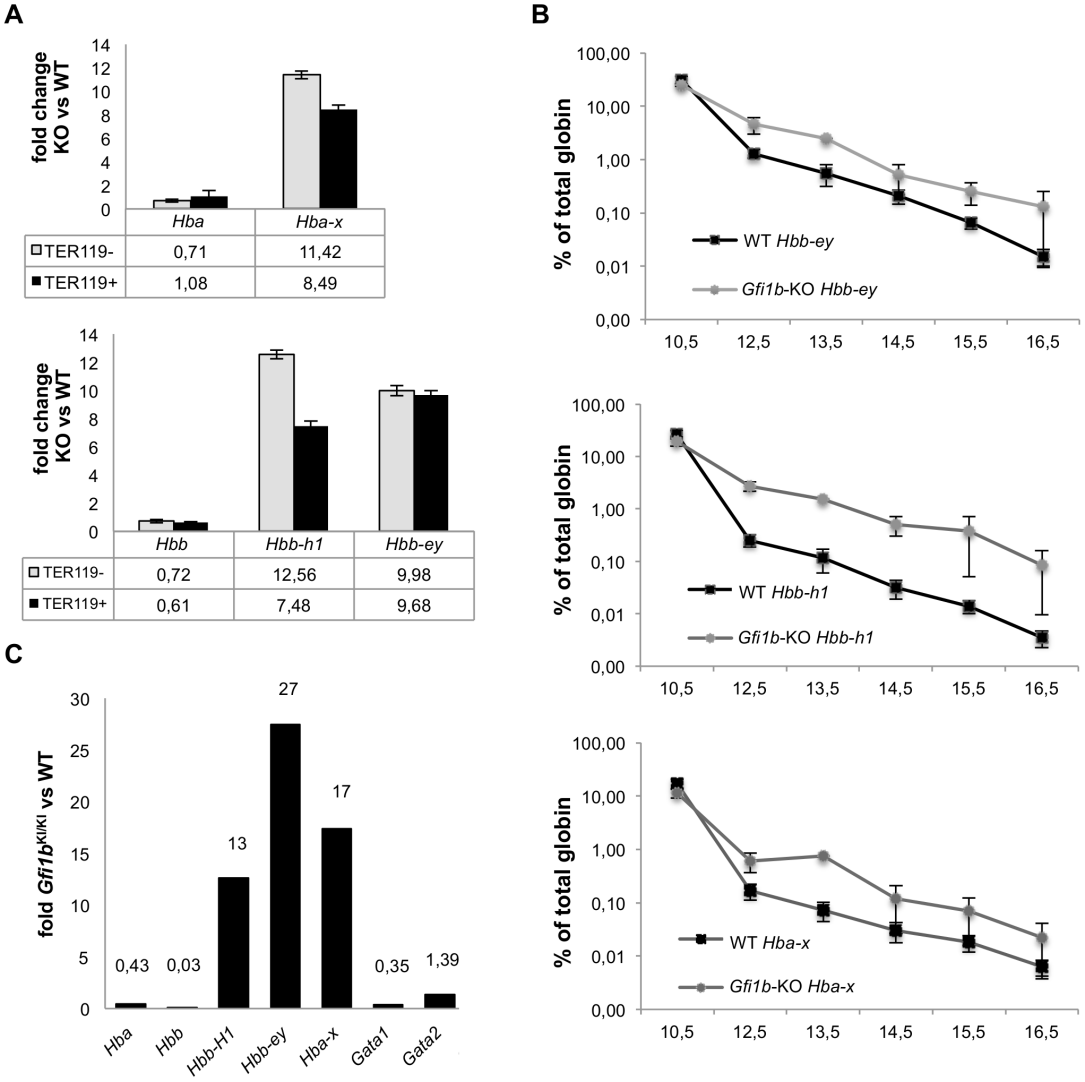
*Gfi1b* deficiency causes delayed and incomplete silencing of embryonic globin genes. A: Q-PCR analysis of embryonic globin gene and *Gata1* expression in CD71^+^, TER119^−^ and CD71^+^, TER119^+^ fetal liver cells from wild type and *EpoR*-Cre induced *Gfi1b* knockout embryos at 15.5 dpc. Bar graphs show the fold change of expression of the indicated genes in *Gfi1b* knockout cells over the wild type expression. Error bars indicate the standard deviation of at least three replicates. B: Line graphs represent the results of the Q-PCR analysis of globin gene expression in fetal liver cells of *EpoR*-Cre, *Gfi1b* ^fl/fl^ (*Gfi1b* KO) or wild type (WT) mice during developmental stages from 10.5 dpc to 16.5 dpc depicted as percent of total globin (logarithmic scale). Error bars represent the standard deviation from triplicate measurements from two (10.5 and 13.5 dpc) to four (12.5, 14.5, 15.5, 16.5 dpc) individuals of each genotype. C: Bar graph representing the results of triplicate Q-PCR analysis of the expression of globin genes, Gata-1 and -2 in *Gfi1b* ^GFP/GFP^ homozygous *Gfi1b* deficient mice compared to heterozygous and wild type littermates at 13.5 dpc. Numbers above bars are fold changes calculated as 2^(Δct KO – Δct WT)^.

**Figure 8:**
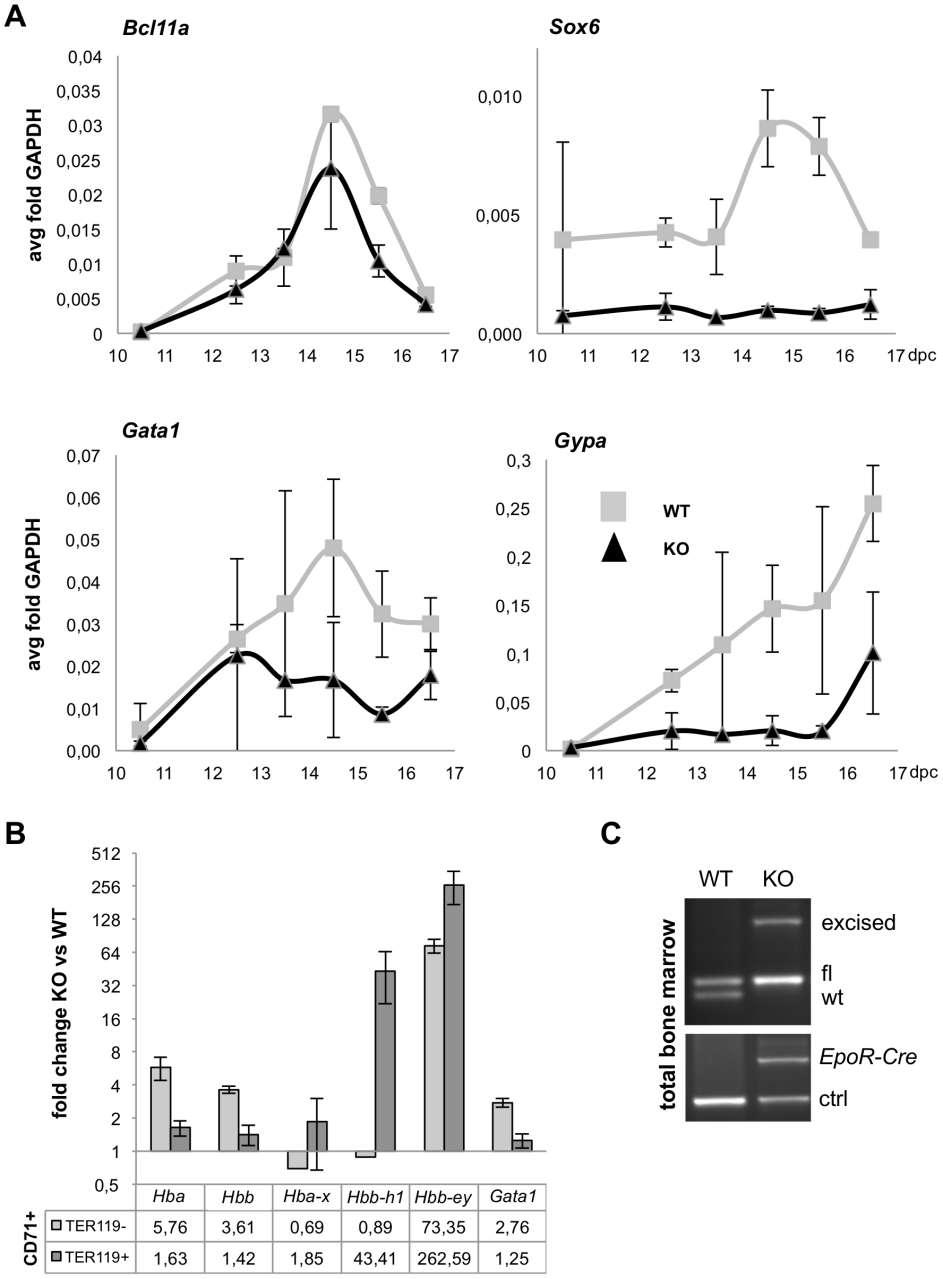
Insufficient activation of *Sox6*, *Gata1* and *Gpa* in *Gfi1b* deficient cells. A: Q-PCR analysis of the relative expression levels of regulators of globin gene expression (*Gata1*), globin gene switch (*Blc11a, Sox6*) and glycophorin A (*Gypa*) normalized to *Gapdh*. Sample sizes were as described in Figure 7B. B: Q-PCR analysis on RNA from CD71^+^, TER119^−^ (pro-erythroblasts) and TER119^+^ (late erythroblasts) live bone marrow cells from a surviving *EpoR*-Cre induced *Gfi1b*-KO mouse compared to a wild type littermate. All measurements were done in triplicates. C: RT-PCR detection of *EpoR*-Cre and *Gfi1b* wt, flox and KO (excised) alleles on total RNA from bone marrow of a surviving mouse with erythroid specific inactivation of *Gfi1b* by *EpoR*-Cre.

To validate a potential regulation of embryonic globin genes by *Gfi1b*, we FACS-sorted CD71^+^, TER119^−/lo^ (proerythroblast) and CD71^+^, TER119^+^ (late erythroblast) fractions of E15.5 fetal liver cells from wt and *EpoR*-Cre *Gfi1b* ^fl/fl^ mice for RT-PCR expression analysis. The expression of the genes for Hba-x, Hbb-bh1 and Hbb-y were up-regulated about 10 fold in *Gfi1b* deficient cells compared to their wt counterparts (Figure 7A). A developmental expression analysis of the *Hba-x*, *Hbb-y* and *Hbb-bh1* genes showed significantly higher levels in *Gfi1b* deficient fetal liver cells than in wild type controls throughout development from stages E12.5 to E16.5 (Figure 7B). A similarly enhanced expression was found in constitutive *Gfi1b* deficient (*Gfi1b* ^GFP/GFP^) fetal liver cells (stage E 13.5) where the expression of *Hbb-bh1*, *Hbb-y* and *Hba-x* was induced up to over 25 fold over controls (Figure 7C). The expression of *Bcl11a*, a target of KLF1, remained similarly regulated during development in *Gfi1b* knockout fetal liver cells and wt controls (Figure 8A). In contrast, *Sox6* was almost absent in *Gfi1b* deficient fetal liver cells at all developmental stages analyzed and *Gata1* was not induced to wt expression levels from stage E 13.5 onwards (Figure 8A). Expression of Glycophorin A (GYPA), an erythroid differentiation marker gene was only induced at a very late stage (E 15.5) in *Gfi1b* deficient cells and never reached wt expression levels (Figure 8A).

To test whether the deregulation of globin genes is also maintained in adult *Gfi1b* deficient mice, we used FACS sorted CD71^+^, TER119^−^ (proerythroblast) or CD71^+^, TER119^+^ (late erythroblast) bone marrow cells from two surviving adult *EpoR*-Cre *Gfi1b* ^fl/fl^ mice for Q-PCR analysis (Figure 8B). Only a partial deletion of the floxed *Gfi1b* allele was detected in TER119^+^ cells (Figure 8C). However, despite this partial deletion, expression of beta-like embryonic hemoglobin genes (*Hbb-y* and *Hbb-bh1*) was still strongly up-regulated in these cells over 60 to over 100 fold, respectively, compared to wild type controls (Figure 8B). *Hba-x* or the adult hemoglobin genes alpha (*Hba*) and beta (*Hbb*) or *Gata1*were only mildly affected by *Gfi1b* deficiency in these cells (Figure 8B). When we compared the effect of *Gfi1b* deficiency on a number of known and suspected regulators of globin gene expression (Figure S8) between fetal liver and adult TER119^+^ bone marrow cells from our array data, the only significant overlap turned out to be a strong overexpression of *Gata2* and of the fetal globin genes themselves. This suggests an important role for *Gata2* in the effect of *Gfi1b* deficiency on fetal globin gene expression and a more direct involvement of GFI1B in the regulation of fetal globin gene expression.

## Discussion

In this study we present evidence that the transcriptional repressor *Gfi1b* is an important factor for murine embryonic and adult definitive erythropoiesis. It has been described previously that *Gfi1b* is highly expressed in megakaryocyte and erythrocyte progenitors (MEPs) and to a lower extent throughout erythrocyte maturation [1]. However a complete study of the role of GFI1B in erythroid differentiation throughout development and in adult stages was hampered by the early embryonic lethality of germline *Gfi1b* knockout mice. We have analyzed three different mouse models, which enabled the deletion of conditional *Gfi1b* alleles either specifically in erythropoiesis at early developmental stages (*EpoR*-Cre mediated) or upon treatment with either pIpC (*Mx*-Cre) or tamoxifen (*Rosa*-Cre-ERT). The results from analyses of all three models indicate that GFI1B is an essential factor required for erythroid maturation during embryonic development in the fetal liver and in adult stages for the production of mature erythroid cells in the bone marrow. This is supported by the reduced frequencies of TER119^+^ erythroblasts that were observed in all *Gfi1b* deficient mice regardless how the ablation was achieved. Analysis of mice 2 and 9 months after a deletion of the conditional *Gfi1b* allele even suggested that *Gfi1b* is absolutely essential to maintain erythropoiesis at long term.

We also found that MEPs are present and even increased in percentage in adult *Gfi1b* deficient mice, which excludes a lack of precursor cells as a the underlying cause for the low frequencies of erythroblasts in the absence of *Gfi1b* and supports a regulatory role of *Gfi1b* during erythroid commitment and development. Probably as a result of the erythroid maturation defect, adult *Gfi1b* deficient mice suffer from anemia as indicated by the low RBC counts, the low hematocrit and hemoglobin levels. In addition, adult *Gfi1b* deficient mice also show extramedullary erythropoiesis, which may be a consequence of the anemia.

Mice in which *Gfi1b* ablation was mediated by the *EpoR*-Cre transgene did not die at midgestation but mainly at birth. This finding points to the possibility that a delayed or inhibited erythroid development is not entirely responsible for the embryonic lethality observed in germline *Gfi1b* knockout mice. However, it cannot be ruled out that incomplete deletion of the floxed *Gfi1b* alleles has allowed enough erythrocytes to mature to allow full development past E13.5–14.5. It remains unclear however, why most *EpoR*-Cre, *Gfi1b* ^fl/fl^ mice die shortly after birth. Additional studies are necessary to clarify this, but a recently described role of the *EpoR* in vascular cells and hypoxic stress [43] may have contributed to this lethality. Different from what was observed in fetal liver, ablation of *Gfi1b* in adult mice, whether erythroid specific or not, did not lead to noticeable accumulation of proerythroblasts (CD71^+^, TER119^lo^ cells) in the bone marrow, but rather to a loss of erythroblast populations (CD71^+^, TER119^+^ cells). It is thus possible that a role of *Gfi1b* in proerythroblast maturation is different in embryonic and adult development.

Our data also demonstrate that *Gfi1b* plays an important role in the regulation of the expression of embryonic globin genes. Regardless how *Gfi1b* ablation was achieved, all animals that lack or are deficient of *Gfi1b* showed a significant increase in embryonic globin gene expression both in fetal liver cells and in bone marrow derived adult erythroid cells. The expression of embryonic globin genes is dependent on fine-tuning by many transcription factors, co-activators and co-repressors. An important role in this regulation has previously been assigned to two complexes, the NF-Y/Gata2 activator hub and the BCL11a/COUPTFII/GATA1 repressor hub that are both present in embryonic and adult erythroid cells on repressed and active y-globin regulatory sequences [44]. The embryonic globin gene repressor function of BCL11a is dependent on the presence of another factor, SOX6, to form long-range interactions [45]. Our experiments showed a strong down-regulation of the expression of both *Sox6* and *Gata1* in *Gfi1b* deficient fetal liver cells and largely unaffected levels of Bcl11a expression. Since SOX6 and GATA1 are both required for a functional repressor complex that occupies the embryonic beta globin locus at regulatory sequences, these findings provide compelling evidence that the deregulation of these two genes is responsible for impaired repression of the embryonic globin genes in *Gfi1b* deficient mice. In contrast, TER119^+^ cells from adult *Gfi1b* knockout mice showed up-regulation of *Gata2* mRNA levels, but almost no change in other known regulators of embryonic globin gene expression. *Gata2* overexpression is known to stimulate fetal globin gene expression[46] and moreover, downregulation of *Gata1* induces *Gata2* expression and results in impaired differentiation of erythroblasts[47], This phenotype is similar to what we observe in our *Gfi1b* knockouts. The analysis of published ChIP-seq data of *Gfi1b* [48] did not reveal a direct occupation of the embryonic globin genes suggesting that *Gfi1b* affects the expression of embryonic globin genes likely via a different mechanism in erythroid cells from fetal liver or adult bone marrow. Although our data clearly establish a role of *Gfi1b* in embryonic globin expression, future studies will have to show whether this occurs through a direct repression of the globin locus by the GFI1B/LSD1/CoREST repressor complex, or whether *Gfi1b* acts indirectly on globin expression possibly in a complex with other regulatory factors.

## Supporting Information

Figure S1 **Analysis of mice from crossings between** ***Gfi1b*** **^fl/fl^ and** ***EpoR*** **-Cre transgenic animals.** A: Newborn mice from a *Gfi1b* ^fl/fl^ x *Gfi1b* ^fl/WT^/*EpoR*-Cre crossing (upper panel). PCR from tail tip DNA identifies floxed or wt *Gfi1b* alleles and the presence of the *EpoR*-Cre transgene (lower panel). The genotype of each pup is given for both *Gfi1b* and the *EpoR*-Cre transgene. B: PCR analysis of recombination of the *Gfi1b* allele in fetal liver cells from two littermates from a *Gfi1b* ^fl/fl^ x *Gfi1b* ^fl/WT^/*EpoR*-Cre crossing at 14.5 dpc (upper panel). The different alleles detected are indicated; only in the presence of *EpoR*-Cre the recombined knockout *Gfi1b* allele is detected. Recombination of the floxed *Gfi1b* allele is incomplete, which is possibly due to the presence of non-erythroid cells in fetal liver, but more likely is a consequence of a specific selection for non *Gfi1b* deleted cells during erythropoiesis. C: Although the *Gfi1b*-KO embryos look pale, the fetal livers of these embryos can barely be discriminated from wt fetal livers.
(TIF)

Figure S2 **RT-PCR analysis of tissues and cells from** ***Mx*** **-Cre,** ***Gfi1b*** **^fl/fl^ transgenic mice.** An efficient, but not complete recombination of the floxed *Gfi1b* alleles was detected by RT-PCR using FACS sorted TER119^+^ cells or total bone marrow (BM), spleen (Sp) or thymus (Thy).
(TIF)

Figure S3 **Analysis of spleens from** ***Mx*** **-Cre,** ***Gfi1b*** **^fl/fl^ animals after pIpC induced deletion.** A: Spleens and normalized spleen weight from animals with the indicated genotype. B: Flow cytometric analysis of splenocytes from the indicated animals for the markers CD71 and TER119.
(TIF)

Figure S4 RT-PCR analysis of bone marrow cells from *Rosa*-Cre-ERT, *Gfi1b* ^fl/fl^ and *Rosa*-Cre-ERT, *Gfi1b* ^wt/fl^ transgenic mice. Complete recombination of the floxed *Gfi1b* alleles was detected by RT-PCR in *Rosa*-Cre-ERT *Gfi1b* ^wt/fl^ mice upon tamoxifen treatment.
(TIF)

Figure S5 **Effect of tamoxifen mediated ablation of** ***Gfi1b*** **in adult** ***Rosa*** **-Cre-ERT,** ***Gfi1b*** **^fl^ mice.** A: Flow cytometric analysis of cells from the indicated mice to detect different erythroblast cell populations according to CD71 and TER119 marker expression. (B) Schema of Tamoxifen treatment. Mice were analyzed 2–8 days after receiving two IP injections of tamoxifen in two days (100 mg/kg the first day and 50 mg/kg the second day). (C) 4 to 7 mice were analyzed for both genotypes at all time points and plotted as mean ± SD for the four erythroblast cell populations.
(TIF)

Figure S6 **Long term effect of tamoxifen mediated ablation of** ***Gfi1b*** **in adult** ***Rosa*** **-Cre-ERT,** ***Gfi1b*** **^fl/fl^ mice.** A: Flow cytometric analysis of cells from the indicated mice to detect different erythroblast cell populations according to CD71 and TER119 marker expression. Mice were analyzed 2 months after tamoxifen treatment as described in Figure S5. B: Quantification of the frequency of the indicated cell subsets from the mice characterized in (A). Proerythroblast: CD71^+^ TER119^lo/−^, Basophilic erythroblasts: CD71^+^, TER119^+^, Polychromatophilic erythroblasts: CD71^med^, TER119^+^, Orthochromatophilic erythroblasts: CD71^lo^, TER119^+^. C: PCR analysis of DNA from total bone marrow from *Rosa*-Cre-ERT, *Gfi1b* ^GFP/fl^ or *Rosa*-Cre-ERT, *Gfi1b* ^fl/fl^ mice to detect the wt, floxed or excised (KO) alleles.
(TIF)

Figure S7 **Box-and-Whisker plot of gene level normalized intensity for** ***Gfi1b*** **in wt and** ***Gfi1b*** **-KO fetal liver cells.** The upper plot shows smallest value, first quantile, median, third quantile and largest value of the *Gfi1b*-gene level normalized intensities of wild type (red) and *Gfi1b* knockout (blue) TER119^+^ fetal liver cells analyzed in duplicates on Affymetrix gene-1.0-ST arrays that allow for exon-level analysis. Exons 10481308, 1048130 and 10481310 (including first ATG) are bordered with loxP sites in the conditional *Gfi1b*-KO and should be deleted by CRE recombination. This is a proof for the deletion of *Gfi1b* by *EpoR*-Cre in TER119^+^ fetal liver cells. The lower plot does show the exon-intron structure and gene-1.0ST array probesets covering the *Gfi1b*-gene and analyzed here. Both plots were generated using the web-tool “Gene array analyzer” (http://gaa.mpi-bn.mpg.de/) [49].
(TIF)

Figure S8 **Change of expression of globin genes and their regulators induced by** ***Gfi1b*** **deficiency.** A: Scatter plot demonstrating the relation of the magnitude of gene expression changes induced by *Gfi1b* deficiency in fetal liver cells at day 14.5 relative to the probability of a significant change of expression (rawp). Values were taken from array data sets described in Figure S7. Genes visualized are either globin genes or known or suspected regulators of globin gene expression. Labels represent the official gene symbols and dots represent the data for gene level analysis of array data. B: Bar graph representing the magnitude of gene expression changes induced by *Gfi1b* deficiency in TER119^+^ bone marrow cells from adult mice as measured on affymetrix MOE430-2 expression arrays. Data are from single array experiments, not allowing for p-value determination. The same genes as in (A) were analyzed. Multiple probesets for single genes were averaged. Gene expression changes are indicated in log-scale. Dotted lines indicate the levels of 1.5-fold or 2-fold changes in gene expression level as indicated.
(TIF)
