# Supplementary figures and images for: Growth Factor Independence 1b (Gfi1b) Is Important for the Maturation of Erythroid Cells and the Regulation of Embryonic Globin Expression
Source: PLoS One. 2014 May 6;9(5):e96636. doi: 10.1371/journal.pone.0096636 (PMC4011847; doi:10.1371/journal.pone.0096636)

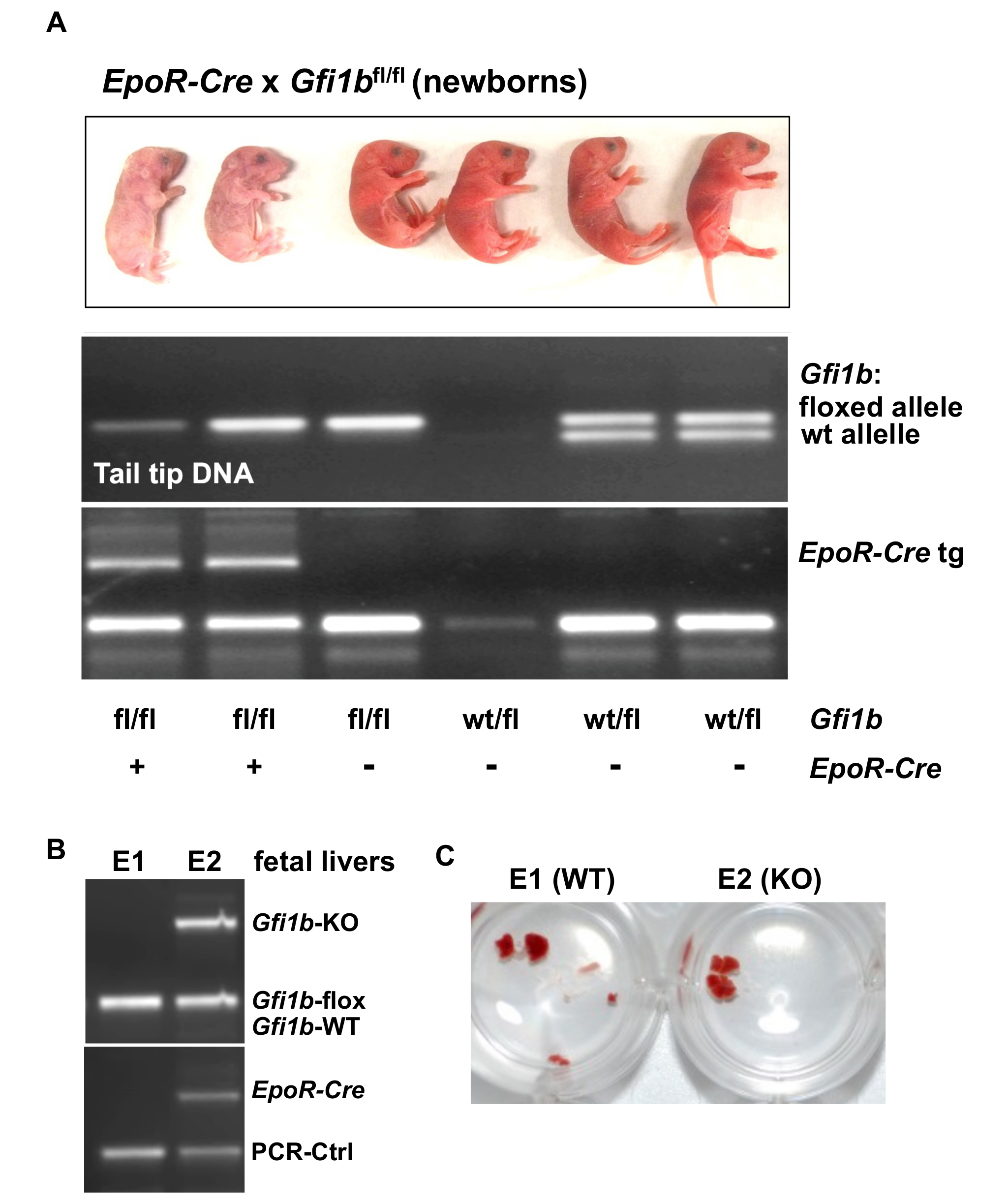

Supplement: Figure S1 — Analysis of mice from crossings between Gfi1b fl/fl and EpoR -Cre transgenic animals. A: Newborn mice from a Gfi1b fl/fl x Gfi1b fl/WT/EpoR-Cre crossing (upper panel). PCR from tail tip DNA identifies floxed or wt Gfi1b alleles and the presence of the EpoR-Cre transgene (lower panel). The genotype of each pup is given for both Gfi1b and the EpoR-Cre transgene. B: PCR analysis of recombination of the Gfi1b allele in fetal liver cells from two littermates from a Gfi1b fl/fl x Gfi1b fl/WT/EpoR-Cre crossing at 14.5 dpc (upper panel). The different alleles detected are indicated; only in the presence of EpoR-Cre the recombined knockout Gfi1b allele is detected. Recombination of the floxed Gfi1b allele is incomplete, which is possibly due to the presence of non-erythroid cells in fetal liver, but more likely is a consequence of a specific selection for non Gfi1b deleted cells during erythropoiesis. C: Although the Gfi1b-KO embryos look pale, the fetal livers of these embryos can barely be discriminated from wt fetal livers. (TIF) [file pone.0096636.s001.tif]

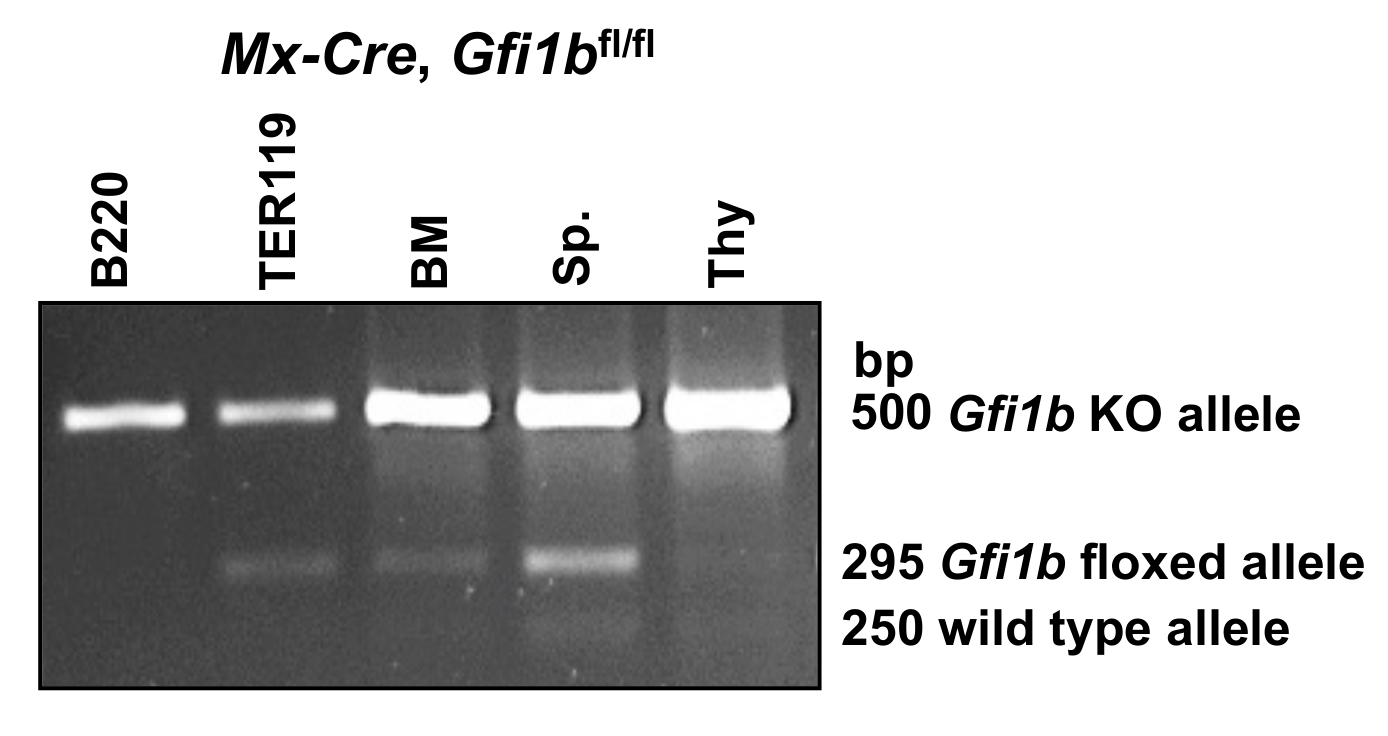

Supplement: Figure S2 — RT-PCR analysis of tissues and cells from Mx -Cre, Gfi1b fl/fl transgenic mice. An efficient, but not complete recombination of the floxed Gfi1b alleles was detected by RT-PCR using FACS sorted TER119+ cells or total bone marrow (BM), spleen (Sp) or thymus (Thy). (TIF) [file pone.0096636.s002.tif]

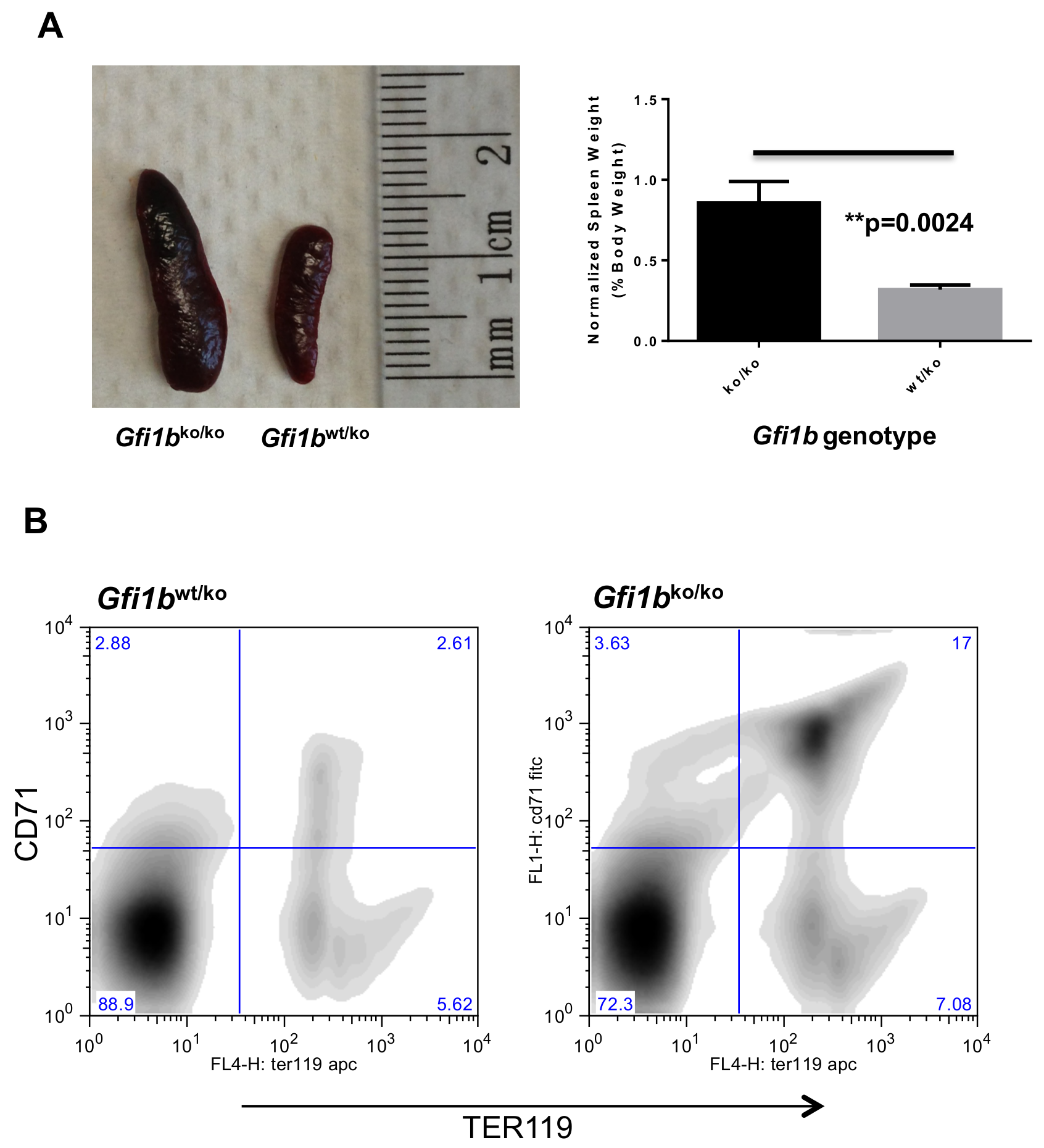

Supplement: Figure S3 — Analysis of spleens from Mx -Cre, Gfi1b fl/fl animals after pIpC induced deletion. A: Spleens and normalized spleen weight from animals with the indicated genotype. B: Flow cytometric analysis of splenocytes from the indicated animals for the markers CD71 and TER119. (TIF) [file pone.0096636.s003.tif]

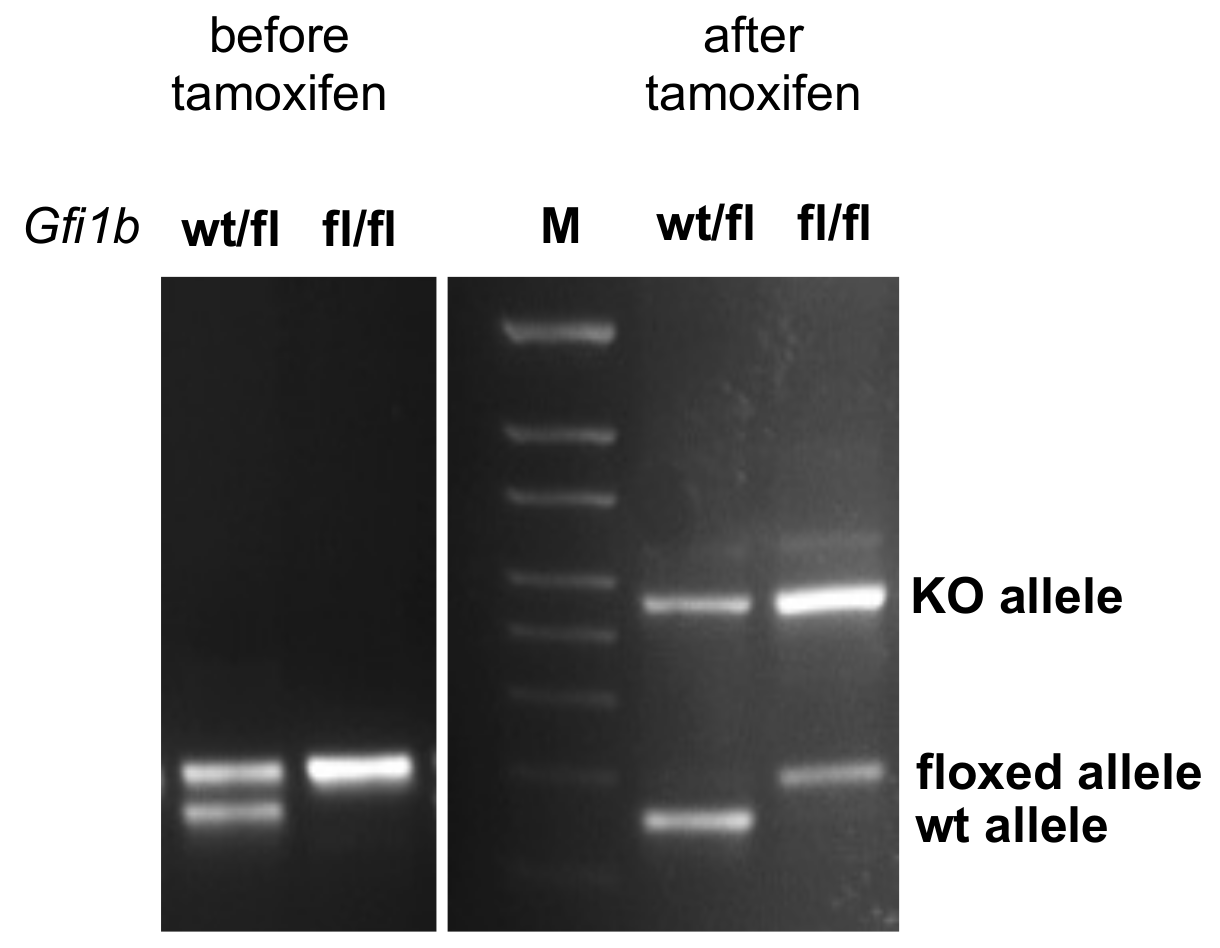

Supplement: Figure S4 — RT-PCR analysis of bone marrow cells from Rosa-Cre-ERT, Gfi1b fl/fl and Rosa-Cre-ERT, Gfi1b wt/fl transgenic mice. Complete recombination of the floxed Gfi1b alleles was detected by RT-PCR in Rosa-Cre-ERT Gfi1b wt/fl mice upon tamoxifen treatment. (TIF) [file pone.0096636.s004.tif]

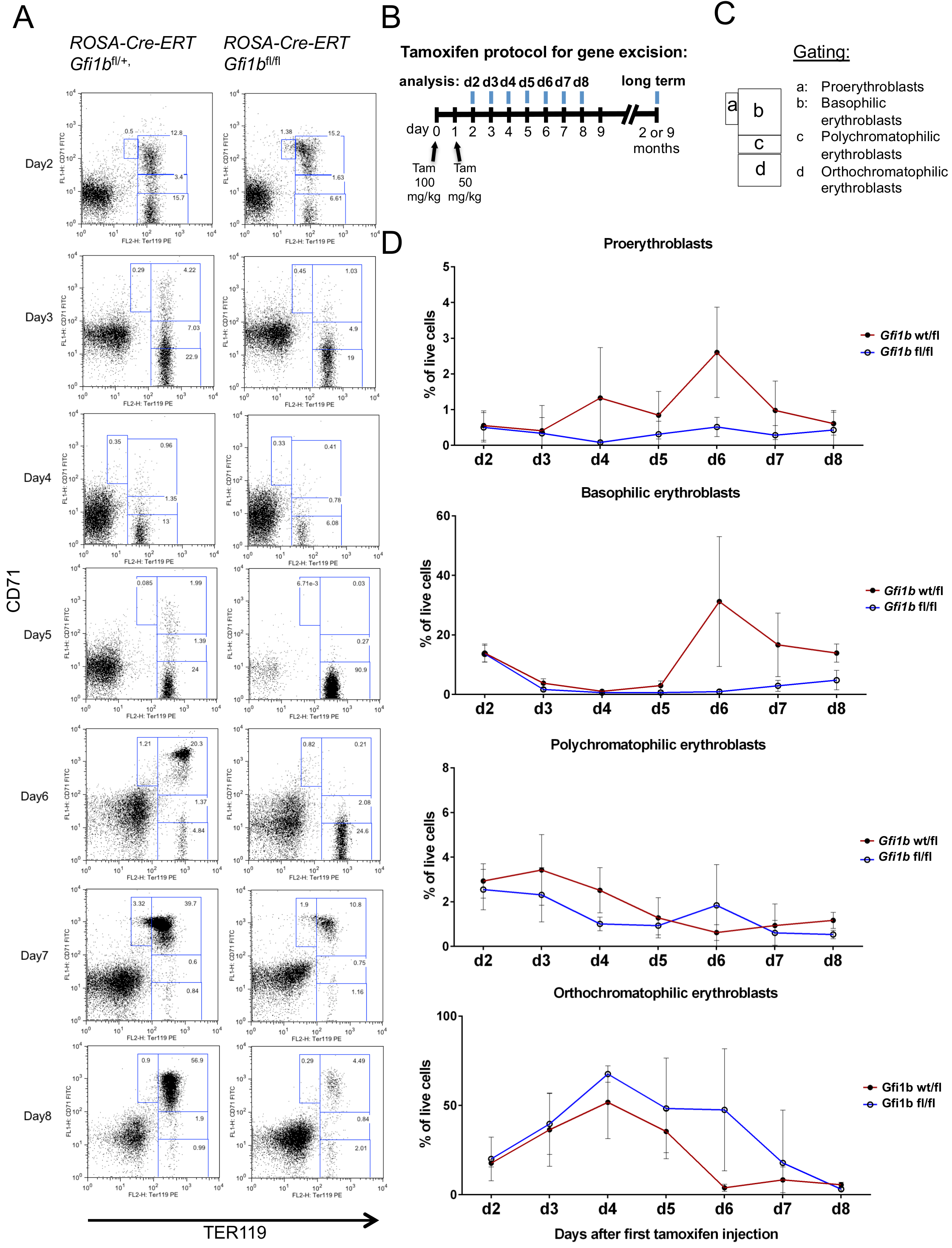

Supplement: Figure S5 — Effect of tamoxifen mediated ablation of Gfi1b in adult Rosa -Cre-ERT, Gfi1b fl mice. A: Flow cytometric analysis of cells from the indicated mice to detect different erythroblast cell populations according to CD71 and TER119 marker expression. (B) Schema of Tamoxifen treatment. Mice were analyzed 2–8 days after receiving two IP injections of tamoxifen in two days (100 mg/kg the first day and 50 mg/kg the second day). (C) 4 to 7 mice were analyzed for both genotypes at all time points and plotted as mean ± SD for the four erythroblast cell populations. (TIF) [file pone.0096636.s005.tif]

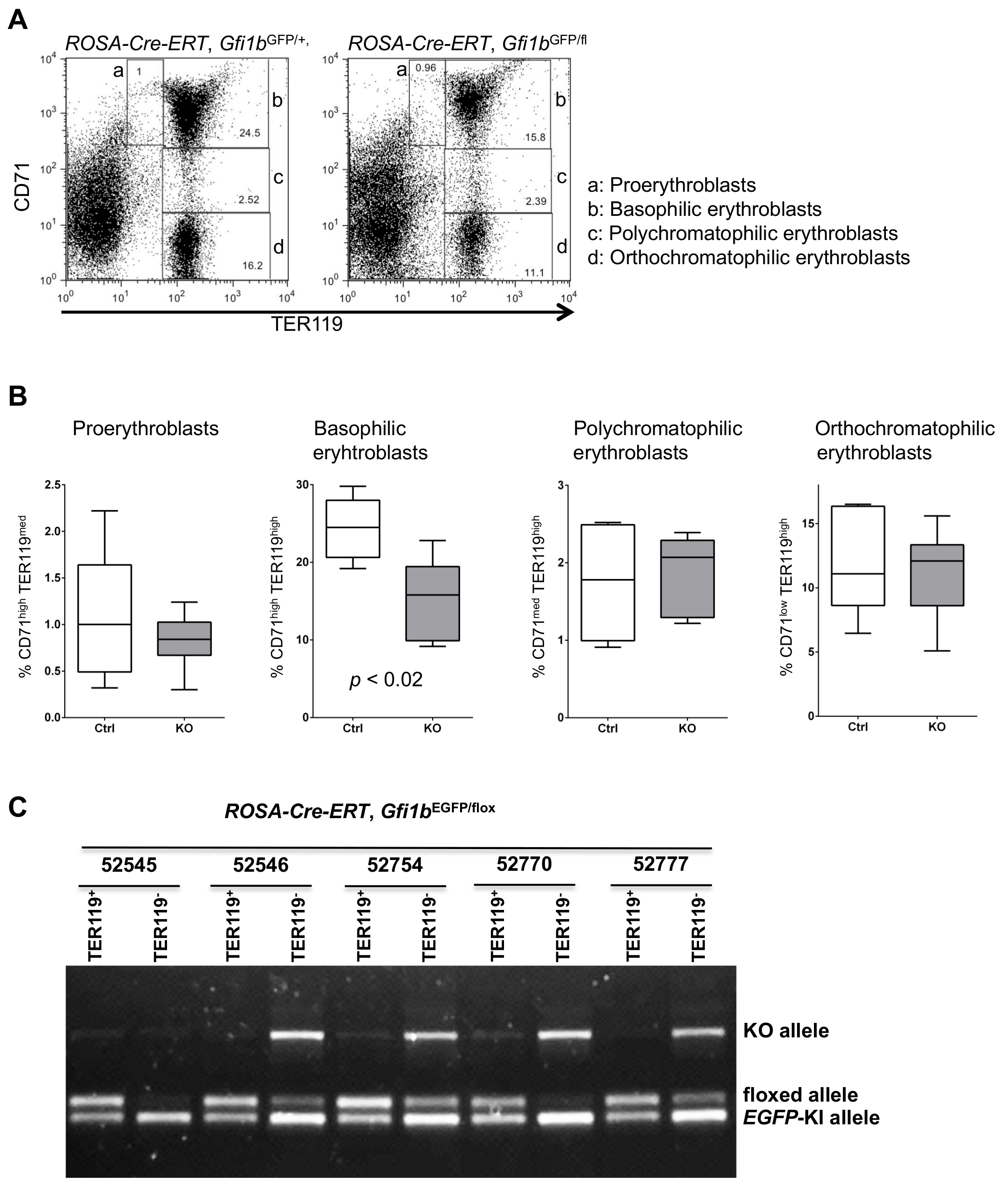

Supplement: Figure S6 — Long term effect of tamoxifen mediated ablation of Gfi1b in adult Rosa -Cre-ERT, Gfi1b fl/fl mice. A: Flow cytometric analysis of cells from the indicated mice to detect different erythroblast cell populations according to CD71 and TER119 marker expression. Mice were analyzed 2 months after tamoxifen treatment as described in Figure S5. B: Quantification of the frequency of the indicated cell subsets from the mice characterized in (A). Proerythroblast: CD71+ TER119lo/−, Basophilic erythroblasts: CD71+, TER119+, Polychromatophilic erythroblasts: CD71med, TER119+, Orthochromatophilic erythroblasts: CD71lo, TER119+. C: PCR analysis of DNA from total bone marrow from Rosa-Cre-ERT, Gfi1b GFP/fl or Rosa-Cre-ERT, Gfi1b fl/fl mice to detect the wt, floxed or excised (KO) alleles. (TIF) [file pone.0096636.s006.tif]

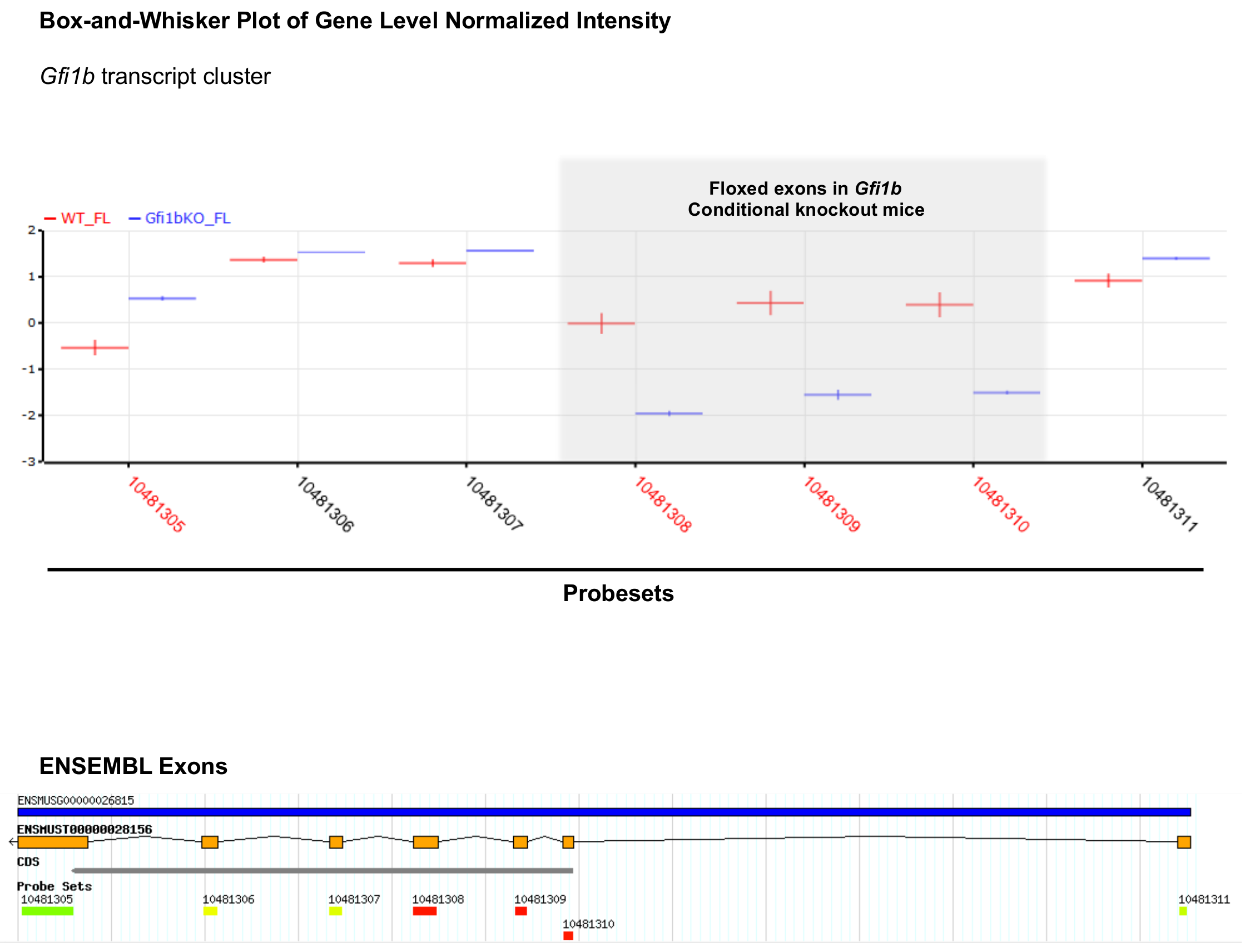

Supplement: Figure S7 — Box-and-Whisker plot of gene level normalized intensity for Gfi1b in wt and Gfi1b -KO fetal liver cells. The upper plot shows smallest value, first quantile, median, third quantile and largest value of the Gfi1b-gene level normalized intensities of wild type (red) and Gfi1b knockout (blue) TER119+ fetal liver cells analyzed in duplicates on Affymetrix gene-1.0-ST arrays that allow for exon-level analysis. Exons 10481308, 1048130 and 10481310 (including first ATG) are bordered with loxP sites in the conditional Gfi1b-KO and should be deleted by CRE recombination. This is a proof for the deletion of Gfi1b by EpoR-Cre in TER119+ fetal liver cells. The lower plot does show the exon-intron structure and gene-1.0ST array probesets covering the Gfi1b-gene and analyzed here. Both plots were generated using the web-tool “Gene array analyzer” (http://gaa.mpi-bn.mpg.de/) [49]. (TIF) [file pone.0096636.s007.tif]

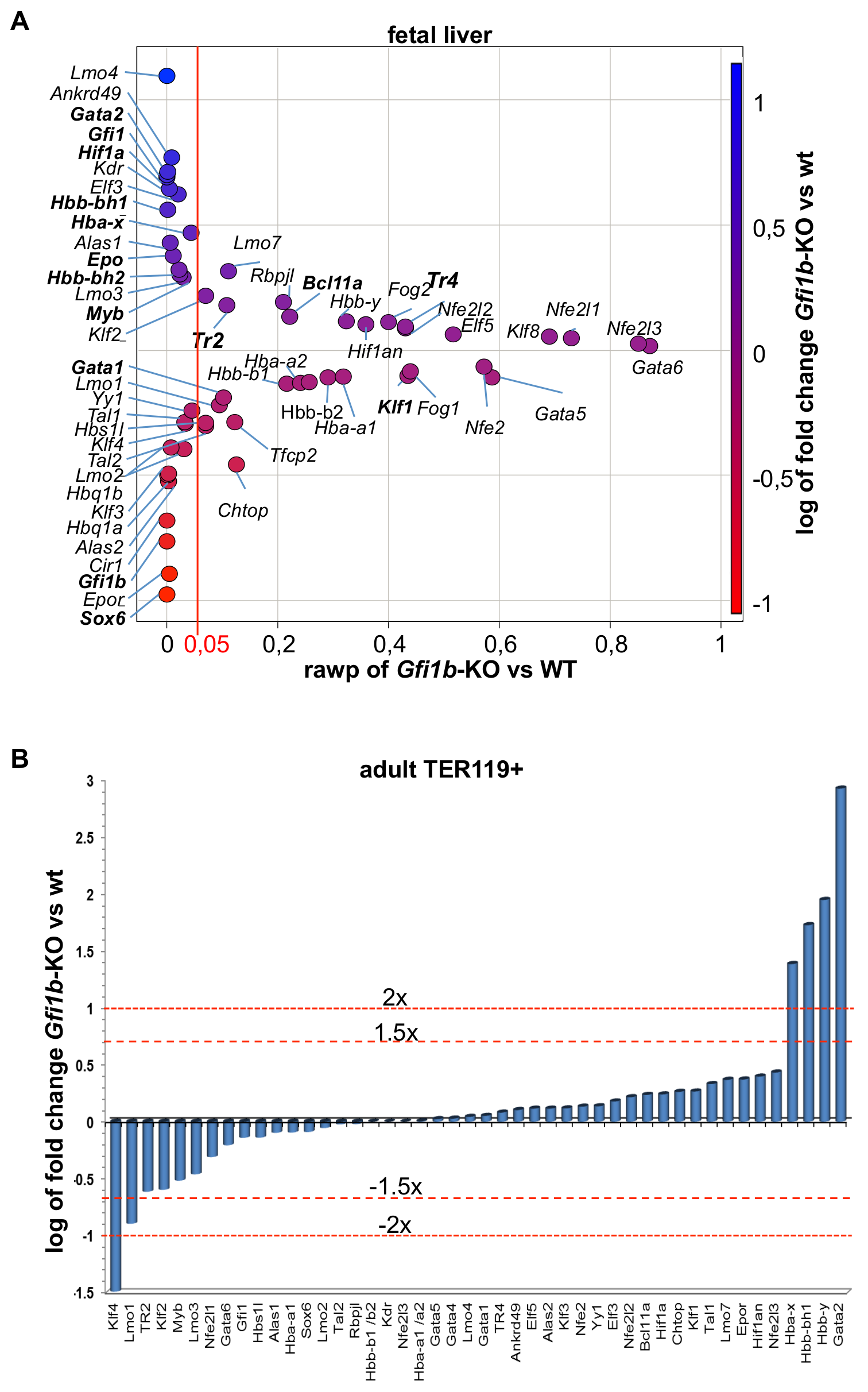

Supplement: Figure S8 — Change of expression of globin genes and their regulators induced by Gfi1b deficiency. A: Scatter plot demonstrating the relation of the magnitude of gene expression changes induced by Gfi1b deficiency in fetal liver cells at day 14.5 relative to the probability of a significant change of expression (rawp). Values were taken from array data sets described in Figure S7. Genes visualized are either globin genes or known or suspected regulators of globin gene expression. Labels represent the official gene symbols and dots represent the data for gene level analysis of array data. B: Bar graph representing the magnitude of gene expression changes induced by Gfi1b deficiency in TER119+ bone marrow cells from adult mice as measured on affymetrix MOE430-2 expression arrays. Data are from single array experiments, not allowing for p-value determination. The same genes as in (A) were analyzed. Multiple probesets for single genes were averaged. Gene expression changes are indicated in log-scale. Dotted lines indicate the levels of 1.5-fold or 2-fold changes in gene expression level as indicated. (TIF) [file pone.0096636.s008.tif]
